# “Textual Prosody” Can Change Impressions of Reading in People With Normal Hearing and Hearing Loss

**DOI:** 10.3389/fpsyg.2020.548619

**Published:** 2020-12-17

**Authors:** Miki Uetsuki, Junji Watanabe, Kazushi Maruya

**Affiliations:** ^1^Department of Community Studies, Aoyama Gakuin University, Kanagawa, Japan; ^2^Communication Science Laboratories, Nippon Telegraph and Telephone Corporation, Kanagawa, Japan

**Keywords:** hearing loss, dynamic text presentation, reading, human interface, textual prosody

## Abstract

Recently, dynamic text presentation, such as scrolling text, has been widely used. Texts are often presented at constant timing and speed in conventional dynamic text presentation. However, dynamic text presentation enables visually presented texts to indicate timing information, such as prosody, and the texts might influence the impression of reading. In this paper, we examined this possibility by focusing on the temporal features of digital text in which texts are represented sequentially and with varying speed, duration, and timing. We call this “textual prosody.” We used three types of textual prosody: “Recorded,” “Shuffled,” and “Constant.” Recorded prosody is the reproduction of a reader’s reading with pauses and varying speed that simulates talking. Shuffled prosody randomly shuffles the time course of speed and pauses in the recorded type. Constant prosody has a constant presentation speed and provides no timing information. Experiment 1 examined the effect of textual prosody on people with normal hearing. Participants read dynamic text with textual prosody silently and rated their impressions of texts. The results showed that readers with normal hearing preferred recorded textual prosody and constant prosody at the optimum speed (6 letters/second). Recorded prosody was also preferred at a low presentation speed. Experiment 2 examined the characteristics of textual prosody using an articulatory suppression paradigm. The results showed that some textual prosody was stored in the articulatory loop despite it being presented visually. In Experiment 3, we examined the effect of textual prosody with readers with hearing loss. The results demonstrated that readers with hearing loss had positive impressions at relatively low presentation speeds when the recorded prosody was presented. The results of this study indicate that the temporal structure is processed regardless of whether the input is visual or auditory. Moreover, these results suggest that textual prosody can enrich reading not only in people with normal hearing but also in those with hearing loss, regardless of acoustic experiences.

## Introduction

Dynamic text presentation is used in everyday life, such as in electronic advertisements and TV tickers. Text scrolling within a fixed speed and direction is often used to show a larger amount of information in a limited space. In addition, text flashing is sometimes used to catch the audience’s attention. The presentation rate of dynamic text is an important factor for readers, as they cannot control the speed in most conventional dynamic text presentation. When the presentation speed is too high, the reading performance becomes poor (Juola et al., 1982; Potter, 1984; Miyake et al., 1994; Wang and Kan, 2004; Bélanger et al., 2012). On the other hand, very low speeds also result in poor reading performance (Legge et al., 1989), as the rhythm in reading is lost because readers cannot extract information beyond individual words (Gibson and Levin, 1975, p. 539). Uetsuki et al. (2017) demonstrated that there is an optimum speed of dynamic text with a rate similar to the oral reading rate of news readers. Chujyo et al. (1993) and Morita et al. (2007) also found that the larger the number of characters displayed, the faster and more comfortable is the speed. This means that when the size of the presentation window is large, the information obtained from the peripheral vision is also utilized to read scrolling texts while chunking appropriately.

Though dynamic text is useful, conventional dynamic text cannot convey complex paralinguistic information, such as emotions, intonations, speaker’s speed, and duration that can be conveyed by spoken language. While reading silently, we often have a subjective experience of inner speech, which resembles overt speech (Filik and Barber, 2011). Hirose (2003), Ashby and Clifton (2005), Ashby (2006), and Hirotani et al. (2006) reported prosodic processing while reading texts silently. For example, Hirotani et al. (2006) demonstrated that readers pause at punctuation marks during silent reading, suggesting that intonation boundaries and punctuation are associated. Instead, explicit prosody, which is defined as an intrinsic feature of spoken language concerned with phonetic features including intonation, rhythm, pauses, and speed of a speech utterance (Cutler et al., 1997), is one of the most powerful ways to convey paralinguistic information in spoken language. It contains useful information for communicating emotional states and the intentions of speakers beyond linguistic representations. While we can use complex temporal structures (timing information) to add paralinguistic information to spoken language, the temporal structure used in dynamic text presentation is limited to relatively simple ways such as kinetic typography and animated texts. Even with those simple temporal structures, some researchers have noted the possibility that those structures can convey emotionality (e.g., Wong, 1996; Malik et al., 2009). Concerning visual temporal structures, Potter (1984, p. 91) pointed out the possibility that when letters appear sequentially and the speed and timing of their appearance are changed, the temporal information conveyed by the dynamic text presentation might play a similar role as the prosody in the spoken language. If dynamic text presentation contains complex and appropriate temporal structures, the added paralinguistic information may enhance our reading. In other words, reading might become smoother or impressions of reading might be enriched by the temporal structure of visually presented texts.

This paper addresses three questions. First, we focus on whether impressions of reading are affected, as they are with prosody, by adding visual timing information (i.e., varying the speed and timings of pauses) to the written language. We call this “textual prosody.” The temporal structures in conventional dynamic text presentation (e.g., scrolling with a constant speed) do not convey timing information. For our purpose, we adopted a special dynamic text presentation format to enable textual prosody. In this format, the letters are statically displayed, but the contrast of letters changes dynamically (Maruya et al., 2012, 2013; Uetsuki et al., 2017). For example, when the characters are displayed, their contrast increases from zero over 2 s, stays at the maximum contrast level for a second, and decreases to zero over 2 s. In other words, the letters appeared gradually, remain at high contrast for a while, and disappear gradually. In addition, we recorded the reading speeds of one example reader at each text location and modulated the speed of text appearance based on the recorded reading speed. The complex temporal profile based on actual human behaviors may give a sense of animacy, a feeling that something living is present and behaving with a particular intention (Heider and Simmel, 1944; Michotte, 1963; Dasser et al., 1989; Tremoulet and Feldman, 2000; Gao et al., 2010) and affects the reader’s impression of content (Maruya et al., 2013). For example, Maruya et al. (2013) demonstrated that readers have warmer and softer impressions when texts were presented at a low speed.

Our second research question was whether information conveyed by textual prosody is stored visually or auditorily. Although texts are presented visually, they may convey temporal information as prosody. Normally, auditory prosody information is initially processed through the listener’s ears and stored auditorily. On the other hand, textual prosody is initially processed through the reader’s eyes. It is not clear whether the information conveyed by textual prosody is stored visually or auditorily. To determine this, we examined the characteristics of textual prosody using an articulatory suppression paradigm. Articulatory suppression is a research tool that is often used to explore phonological processing in reading (Morita and Saito, 2007; Leinenger, 2014). In the working memory framework of Baddeley and Hitch (Baddeley and Hitch, 1974; Baddeley, 1986; Norris et al., 2018), the articulatory loop performs subvocal rehearsal and record written input into a phonological form that can be retained in the phonological store. Articulatory suppression prevents the articulatory loop selectively (Baddeley, 1986). Auditory material has an obligatory access to the phonological store, whereas only a part of visually presented information will enter the phonological store (Hanley and Bakopoulou, 2003). Filik and Barber (2011) demonstrated that articulatory phonology activated during sentence reading contains readers’ accents. If suppression interferes with a reading task, phonological coding is assumed to be necessary or at least a part of the task under investigation (Leinenger, 2014).

Finally, we asked whether the effect of textual prosody depends on acoustic experiences in daily life. People with hearing loss exhibit problems in learning to read as a result of the difficulties they face in developing spoken language (Gallego et al., 2016). There seems to be a sensitive period in early postnatal life, during which the brain is highly efficient in establishing connections between the auditory input of speech and the development of linguistic skills (Kuhl et al., 2005; Markman et al., 2011; Gallego et al., 2016). The higher the sound deprivation in the initial years of life, the greater is the negative impact on the maturation of auditory pathways and reading comprehension (Connor and Zwolan, 2004; Sainz and de la Torre, 2005; Gallego et al., 2016). In regard to the language processing of readers with hearing loss, however, Hanson and Fowler (1987) demonstrated that when the participants were asked to judge whether the simultaneously presented two letter strings were English words or not (a lexical decision task), both the normal hearing and hearing loss readers could respond faster to rhyming word pairs (e.g., MARK-DARK, LOAD-TOAD, DONE-NONE, and SAVE-WAVE) than to non-rhyming word pairs (e.g., MARK-TOAD, LOAD-DARK, BONE-GONE, and HAVE-CAVE). This result provided evidence that readers with hearing loss could access phonological information (see Hanson and Fowler (1987) for discussion of the effect of visual similarity). Thus, textual prosody may enrich readings of people with hearing loss.

Sign language is often used as a means of communication in people with hearing loss. Both spoken and signed languages are acquired naturally. Sign language possesses all the linguistic complexity and levels of structure of spoken language (Newman et al., 2010). Spoken and sign languages share many properties, such as phonology (Sandler and Lillo-Martin, 2006; Villameriel et al., 2019). Additionally, sign language exploits sets of regular prosodic features (Morgan et al., 2007), and the prosody of the sign is based on both the timing of the sign’s complete articulation from beginning to end and the fixed ordering of different segments within the movement (Villameriel et al., 2019). Therefore, the effect of textual prosody could be observed if one utilizes their experience of sign language and lip reading to process textual prosody.

In this study, therefore, we examined whether textual prosody can affect impressions of reading (we focus on readability, favorability, and emotionality) of participants with hearing loss in addition to those of participants with normal hearing. If textual prosody can enrich people’s reading, especially in people with hearing loss, it should be a useful tool to convey speech speeds and timing information visually.

We conducted three experiments in which we manipulated the textual prosody of visually presented language and asked readers to judge their impressions of readability, favorability, or emotionality (hereafter, impressions of reading). The textual prosody was manipulated in three ways: recorded, shuffled, and constant prosody. Recorded prosody reproduces utterance speed and duration in correspondence with letters. Shuffled prosody is inappropriate in the sense that the timings of pauses do not match the boundaries of sentences or words. Constant prosody has constant presentation speed and has no prosody. In Experiment 1, we examined the effects of textual prosody, showing visual timing information in readers with normal hearing. If textual prosody affects the impression of reading, the scores of the impressions would be higher/lower than when there is no textual prosody. In Experiment 2, we examined how textual prosody is stored with an articulatory suppression paradigm. If representation of textual prosody is stored in the articulatory loop, scores of impressions of reading under articulatory suppression condition should be worse than under the no suppression condition. In Experiment 3, we examined whether textual prosody affects the impressions of readers with hearing loss. Although people with hearing loss have experience with prosody information in sign language and lip reading, they have less auditory experience. If textual prosody affects the impressions of readers with hearing loss, the scores of the impressions would be higher in recorded prosody than in constant or shuffled prosodies.

## Experiment 1

In this experiment, we presented text to readers with normal hearing at various presentation speeds and with various textual prosodies (timing information of texts). It is assumed that textual prosody may enrich the impression of reading because it offers more paralinguistic information visually. However, prosodic processing occurs even when people read texts silently (Hirose, 2003; Ashby, 2006; Hirotani et al., 2006). If readers adopt their own output of prosodic processing, textual prosody may be ignored and not be utilized. Therefore, we examined whether textual prosody affects the impressions of reading.

### Materials and Methods

#### Participants

This experiment was conducted with two groups to confirm the reproducibility of the results. The first group comprised 29 female college students who volunteered to participate in the experiment. The participants were either in their first or second year in college, and their mean age was 18.62 years (SD: 0.81). The second group comprised 23 female college students who volunteered to participate in the experiment. The participants were either in their second or third year in college, and the mean age of participants was 19.57 years (SD: 0.65). There were no duplicate participants between the first and second groups. The sample size of each group was determined based on prior studies (Maruya et al., 2012, 2013; Uetsuki et al., 2017). There were no participants with hearing loss. This experiment was performed in compliance with the Declaration of Helsinki. The protocol was approved by the Ethics Committee of Hakodate Junior College (approval number: H21-02) or by the Ethics Committee of Aoyama Gakuin University (approval number: Ao18–5). This study was carried out in accordance with the recommendations of Provisions of Experiments, Ethics Committee of Hakodate Junior College and the recommendations of Aoyama Gakuin University Ethics Committee for human research with written informed consent from all participants.

#### Dynamic Text Presentation Format

**FIGURE 1:**
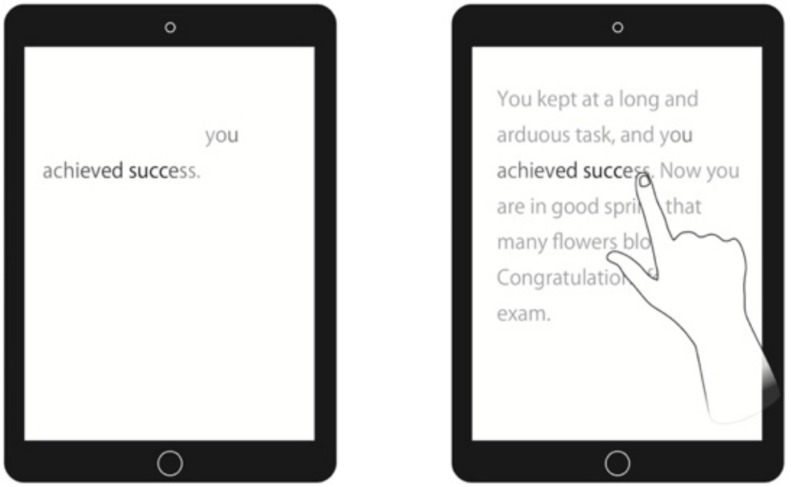
Dynamic text presentation format. In automatic mode, letters appear on the white background with a constant temporal interval (left). In tracing mode, users can trace letters that are barely visible at initial display (right) and replay it on the white background (left).

We adapted a special dynamic text presentation format to present textual prosody described in the introduction section. To measure, record, and present reading positions, we utilized a computer program “Yu bi Yomu” on tablet devices (Maruya et al., 2012, 2013; Uetsuki et al., 2017). In this software, onscreen text is barely visible at the initial display and, when a user touches the panel, the contrast of the letter at finger position increases and then decreases (right illustration in Figure 1). If the user traces the sentence from the beginning, the sentences will appear and disappear sequentially. The user traces characters according to his/her reading. For example, if the user reads a word slowly, they trace the word slowly. This mode is called “Tracing mode.” In addition to this function, the software can present letters so that the timing of contrast change for each letter shifts at constant temporal intervals. The letters appear and disappear as if the contrast change moves with a constant speed (left illustration in Figure 1). This mode is called “Automatic mode.” We made stimuli video using this software and we presented texts in automatic mode and video recorded in tracing mode in our experiment.

#### Textual Prosody (Visual Timing Information)

We also created three types of textual prosodies (hereafter, “prosody type”) for each presentation speed using the software. One of these types of textual prosodies is “recorded.” This type uses the tracing mode. One of the authors traced the sentences using pauses and changing the tracing speed as if talking. The tracing movement is assumed to exhibit similar speed and timing as that of their actual or inner speech. The software “Yu bi Yomu” can record a video of how the letters appear and disappear and replay it without barely visible letters at initial presentation. Here, we expected that if the user traces sentences as if talking and replays it, the movement of contrast change might play a role similar to prosody in speech, such as speed and timing. The author read texts with the intention that the meaning of the text would be conveyed correctly. For example, the author paused according to the syllables or large units of text or at the boundaries of some clauses. He/she also read the words that might convey emotions (e.g., “thank you” and “congratulations”) or might be important slower than other words. Only a single reader’s tracing was used as recorded prosody because the more averaged the tracing of multiple readers, the more often the stimulus was displayed at a constant speed. Recorded prosody is appropriate in the sense that the timings of pauses matches the boundaries of a sentence or word. The second type of prosody is “shuffled.” This type randomly shuffles the time course of speed and pauses in the recorded type. Accordingly, pauses occur while presenting a letter, or in the middle of a syllable, a word, or a large unit of text. Shuffled prosody is irrelevant in the sense that the timings of pauses do not match the boundaries of sentences or words. The acceleration of speed is non-zero in recorded and shuffled prosody. The third type of prosody is “constant,” in which the presentation speed is constant and the acceleration of speed is zero. This was achieved in an automatic mode.

#### Presentation Speed

We varied the presentation speed, i.e., 3, 6, or 12 letters/second (hereafter, LPS). This was because previous studies (Price et al., 1996; Uetsuki et al., 2017) showed that the impressions of reading were the most enhanced at 6 LPS. Thus, we used 6 LPS, 3 LPS (1/2 times the speed), and 12 LPS (2 times the speeds). To create three presentation speed conditions for “recorded prosody,” the author adjusted the tracing so that the presentation speed was obtained by dividing the presentation time by the number of letters to create 3, 6, and 12 LPS conditions, respectively. The author tried to read in the same way under three conditions, except for speed (that is, he/she tried to pause or change the speed at the same positions under 3, 6, and 12 LPS conditions). Thus, strictly speaking, recorded prosody is different for the three different presentation speed conditions. We cannot deny the existence of the effects of prosodic variability. However, it is assumed that effects of prosodic variability are much smaller than effects of presentation speed.

#### Text Stimuli

We used four types of Japanese plain text. One was called “Thank you”; it conveys gratitude (“Thank you very much all the time.”). The second one was called “Telegram”; it is a typical telegram that celebrates success on an examination (“You kept at a long and arduous task, and you achieved success. Now you are in a good spring where many flowers bloom. Congratulations for passing the exam.”). The third, “Weather forecast,” presents sentences typical of weather reports on TV news (“Same as yesterday, the area around Japan is in a winter-style air pressure arrangement. It is cloudy in the central city of Tokyo today, and it will rain in some places.”). The forth, “Earthquake warning,” warns of an imminent earthquake and is very familiar to Japanese people (“This is an emergency earthquake flash report. Please beware of a strong shake.”). Japanese text stimuli are in Table A1.

#### Procedures

The software was run on a tablet computer (Apple iPad) and connected to a projector (EPSON Inc., EB-535W). The texts were presented on the projector. The participants were divided into two groups and observed the text stimuli. They sat in three rows, at distances of 2–5 m from the screen. The visual angle of a letter was about 1–2.5°. All participants reported that the stimulus texts were adequately visible.

This experiment was conducted for each text. The nine stimuli (three speeds × three prosody types) per text were presented. It was confirmed that the impression of static text did not change before and after reading the dynamic text repeatedly (Maruya et al., 2013). Thus, the influence of repetition should be small. We presented the stimuli in a randomized order within text stimuli, and the order of trials differed for each participant group. The letters were not visible at first. When a trial started, one of the nine text stimuli was presented, and participants silently read it. After reading, they rated their impressions of the text on a scale from −50 to 50 points (semantic differential method, 100 scales; Osgood et al., 1957; Snider and Osgood, 1969). We measured the impression of reading as readability (readable–unreadable), favorability (like–dislike), and emotionality (emotional–businesslike). Participants rated each impression par trial. The participants evaluated the strength of their impressions of dynamic texts with numerical values. Each condition was repeated twice, and the means of the two trials were used for analysis. This experiment included 72 trials in total (three speeds × three visual prosodies × two repetitions × four texts) and took about 1 h.

### Results

This experiment was conducted with two groups. The data of the two groups were merged because both the groups displayed similar tendencies. Each condition was repeated twice, and the means were calculated as rating values. We also merged the values of the four texts because the tendencies of data in the four texts were similar. A two-way within-subject Analysis of Variance (ANOVA), with prosody type and presentation speed as factors and the rating value as the dependent value, was conducted for each judgment. The degree of freedom was corrected by Greenhouse-Geisser correction when the Mauchly’s sphericity test was found to be significant. When the interaction was significant, we tested simple main effects, using Bonferroni corrections.

**FIGURE 2:**
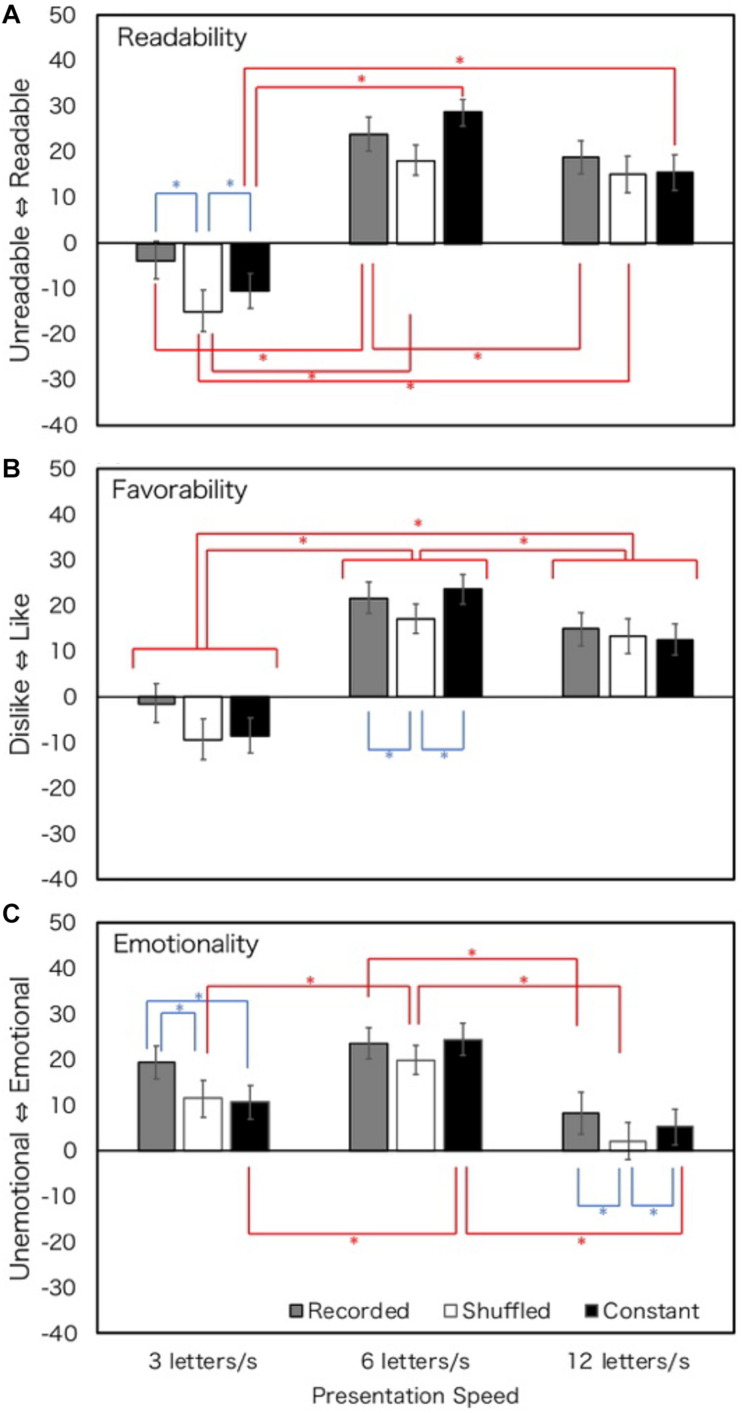
Rating values of Experiment 1 (normal hearing; all texts). Error bars show 95% confidential intervals. Data of four texts were merged. Blue stars show the effect of prosody, and red stars show the effect of presentation speed. ^∗^*p* < 0.05.

The overall results are shown in Figure 2. The main effect of presentation speed and the main effect of prosody type were observed in judgments of favorability. The interactions of speed and prosody type were significant for judgments of readability and emotionality. Table 1 shows the summaries of ANOVA results. Most of the Bonferroni-corrected simple main effects revealed that the impression scores at 6 LPS were higher than those at 3 or 12 LPS, irrespective of prosody types. Participants felt the texts were more readable, favorable, and emotional at 6 LPS. In terms of textual prosody, recorded prosody was consistently more positive than shuffled prosody at 3 and 6 LPS, though not necessarily statistically significant. This suggests that recorded prosody is more readable, favorable and emotional than shuffled prosody under 6 LPS. The difference between recorded and constant prosody was not significant except at 3 LPS in Emotionality.

**TABLE 1:** Results of ANOVA of impression of reading in Experiment 1 (normal hearing; all texts).

Impression	Effect	*F*-value, *p-*value, η*_*p*_*^2^ of main effects, interactions, and simple main effects	Multiple comparison (bonferroni)
Readability	Speed	*F(2, 102) = 122.36, *p* < 0.0001, η*_*p*_*^2^ = 0.71	
	Prosody type	*F(2, 102) = 6.52, *p* = 0.002, η*_*p*_*^2^ = 0.11	
	Speed × Prosody type	*F(4, 204) = 2.66, *p* = 0.034, η*_*p*_*^2^ = 0.05	Recorded: 3 < 12 < 6 LPS
		Simple main effects	Shuffled, Constant: 3 < 6, 12 LPS
		Presentation speed at recorded: *F*(2, 50) = 41.90, *p* < 0.0001, η*_*p*_*^2^ = 0.63	3 LPS: Shuffled < Recorded, Constant
		Presentation speed at shuffled: *F*(2, 50) = 89.58, *p* < 0.0001, η*_*p*_*^2^ = 0.78	
		Presentation speed at constant: *F*(2, 50) = 100.74, *p* < 0.0001, η*_*p*_*^2^ = 0.80	
		Prosody type at 3 LPS: *F*(2, 50) = 10.47, *p* < 0.0001, η*_*p*_*^2^ = 0.30	
		Prosody type at 6 LPS: *F*(2, 50) = 1.35, *p* = 0.2691, η*_*p*_*^2^ = 0.05	
		Prosody type at 12 LPS: *F*(2, 50) = 2.82, *p* = 0.069, η*_*p*_*^2^ = 0.10	
Favorability	Speed	*F(1.62, 82.79) = 134.64, *p* < 0.0001, η*_*p*_*^2^ = 0.73	3 < 12 < 6 LPS
	Prosody type	*F(1.74, 88.89) = 8.98, *p* < 0.0001, η*_*p*_*^2^ = 0.15	Shuffled < Recorded, Constant
	Speed × Prosody type	*F*(3.53, 179.80) = 1.35, *p* = 0.257, η*_*p*_*^2^ = 0.03	
Emotionality	Speed	*F(2, 102) = 25.95, *p* < 0.0001, η*_*p*_*^2^ = 0.34	
	Prosody type	*F(2, 102) = 7.02, *p* = 0.001, η*_*p*_*^2^ = 0.12	
	Speed × Prosody type	*F(4, 204) = 4.48, *p* = 0.002, η*_*p*_*^2^ = 0.08	
		Simple main effects	Recorded: 12 < 6 LPS
		Presentation speed at recorded: *F*(2, 50) = 3.78, *p* = 0.030, η*_*p*_*^2^ = 0.13	Shuffled, Constant: 3, 12 < 6 LPS
		Presentation speed at shuffled: *F*(2, 50) = 27.13, *p* < 0.0001, η*_*p*_*^2^ = 0.52	3 LPS: Shuffled, Constant < Recorded
		Presentation speed at constant: *F*(2, 50) = 19.73, *p* < 0.0001, η*_*p*_*^2^ = 0.44	12 LPS: Shuffled < Recorded, Constant
		Prosody type at 3 LPS: *F*(2, 50) = 11.58, *p* < 0.0001, η*_*p*_*^2^ = 0.2	
		Prosody type at 6 LPS: *F*(2, 50) = 0.73, *p* = 0.485, η*_*p*_*^2^ = 0.03	
		Prosody type at 12 LPS: *F*(2, 50) = 6.01, *p* = 0.005, η*_*p*_*^2^ = 0.19	

*p < 0.05.

### Discussion

In general, the impression of reading at 6 LPS was the highest among readers with normal hearing. This finding is consistent with the optimum reading speed reported by Uetsuki et al. (2017). It is suggested that impressions tend to be the most positive when reading speed is comfortable. As for textual prosody, impressions in recorded prosody tended to be consistently positive compared to those in shuffled prosody at 3 and 6 LPS, although the differences were not necessarily statistically significant. The effect of constant prosody was not different from that of recorded prosody under most conditions. It may be suggested that prosodic processing may occur (Hirose, 2003; Ashby, 2006; Hirotani et al., 2006) when constant prosody is presented, and that the impressions of texts are affected by the reader’s own prosody.

Except for emotionality, the effect of textual prosody was not so clear at 12 LPS, that is, under fast text presentation conditions. The pauses of textual prosody were relatively shorter and the overall speed was faster (the absolute speed difference between relatively slow and fast reading was small) at 12 LPS. The differences between the three textual prosodies may become smaller at 12 LPS because the absolute amount of duration and speed change was the smallest in faster conditions. It is assumed that this is the reason why the effect of textual prosody was not clear at 12 LPS. On the other hand, the rating values in recorded prosody tended to be higher than those in shuffled prosody at 3 and 6 LPS. It is assumed that the effect of recorded prosody was relatively stronger because the absolute duration and speed change were larger at lower speeds. The differences between the textual prosody types were smaller than those observed between the three conditions in presentation speeds.

The rating values of 3, 6, and 12 LPS for each textual prosody in Figure 2 were in the form of an inverted U shape. The kurtoses of the inverted U shape of shuffled and constant prosodies were smaller than those of recorded prosody. However, the kurtosis of the inverted U shape was larger in recorded prosody, and the rating values were high at certain presentation speeds. For emotionality, the peak of the inverted U shape of recorded prosody might be shifted to a slower presentation rate.

In sum, for readers with normal hearing, some enhancement of impression of reading by textual prosody was observed. For recorded prosody, the degradation of impressions at the slow presentation speed could be somehow alleviated. When the presentation speed was appropriate at 6 LPS, presentation with recorded and constant prosody was preferred. When the temporal structure of recorded presentation was shuffled, participants’ evaluations generally were low, which means that the mere presence of acceleration is not sufficient to cause the enhancement by textual prosody and that an appropriate temporal structure is required.

## Experiment 2

Experiment 1 shows that readers with normal hearing prefer text to be presented at the rate of 6 LPS and that textual prosody can affect their impressions of reading. The characteristics of textual prosody, however, are not clear. For example, it is not apparent whether textual prosody is converted to visual or to auditory representations. In this experiment, we examined whether textual prosody is stored as visual or auditory information using an articulatory suppression paradigm. Articulatory suppression prevents the articulatory loop selectively (Baddeley, 1986). If suppression impairs the impression of reading at least one condition, it is assumed that reading texts with textual prosody requires phonological coding.

Although we used three types of prosodies, only recorded prosody is useful and should be utilized because it pauses according to the syllables or large units of text or at the boundaries of some clauses. Constant and shuffled prosody do not need to be stored because constant prosody has no specific prosody and shuffled prosody is irrelevant. Thus, it is assumed that only recorded prosody should be affected by articulatory suppression and that the impressions may be impaired if textual prosody is stored in the articulatory loop. In contrast, if recorded prosody is stored visually and not stored in the articulatory loop, storing the prosody should be easy and the impressions may not be impaired even when participants are articulatory suppressed. We examined this prediction using articulatory suppression and the results revealed the characteristics of textual prosody. Though memory tests are normally used with articulatory suppression, it could not be used because we had presented the same texts repeatedly. We then measured readers’ impressions of texts with articulatory suppression.

### Materials and Methods

#### Participants

Forty-four female college students voluntarily participated in this experiment as a part of their classwork. Sample size was determined by the sample used in Experiment 1. The mean age of the participants was 19.00 years (SD: 0.64). No participants had hearing loss. This experiment was performed in compliance with the Declaration of Helsinki. The protocol was approved by the Ethics Committee of Hakodate Junior College (approval number: H21-02). This study was carried out in accordance with the recommendations of Provisions of Experiments, Ethics Committee of Hakodate Junior College with written informed consent from all participants.

#### Articulatory Suppression

There were two suppression conditions: no suppression and articulatory suppression. In the former condition, participants read the text stimuli silently and had no disturbance. In the latter condition, participants were asked to read text stimuli silently and to repeat “a, i, u, e, o, …” simultaneously.

#### Text Stimuli, Presentation Speed, and Textual Prosody

Text stimuli and three types of textual prosodies were the same as those used in Experiment 1. The presentation speed was fixed at 6 LPS because Experiment 1 showed the impressions of reading were the most positive at that speed.

#### Procedures

The software was run on a tablet computer (Apple iPad) and connected to a projector. Texts were presented on the projector. The participants were divided into two groups and each observed the text stimuli. The visual angle of a letter was about 0.5–2.5°. All participants reported that the stimulus texts were visible well. In one group, an articulatory suppression condition was assigned to the first half of the trials and a non-suppression condition was assigned to the latter half. The order of the suppression conditions was reversed in the second group. In this experiment, we did not include repetition because articulatory suppression was cumbersome for the participants and caused fatigue in them. This experiment included 24 trials (three textual prosodies × four texts × two suppressions) and took about 30 min. Other procedures were the same as those in Experiment 1.

### Results

We merged the values of the four texts because the tendencies of data in the four texts were similar. A two-way within-subject ANOVA, with prosody type and articulatory suppression as factors and the rating value as the dependent value, was conducted for each judgment. The degree of freedom was corrected by Greenhouse-Geisser correction when the Mauchly’s sphericity test was found to be significant. When the interaction was significant, we tested simple main effects using Bonferroni corrections.

**FIGURE 3:**
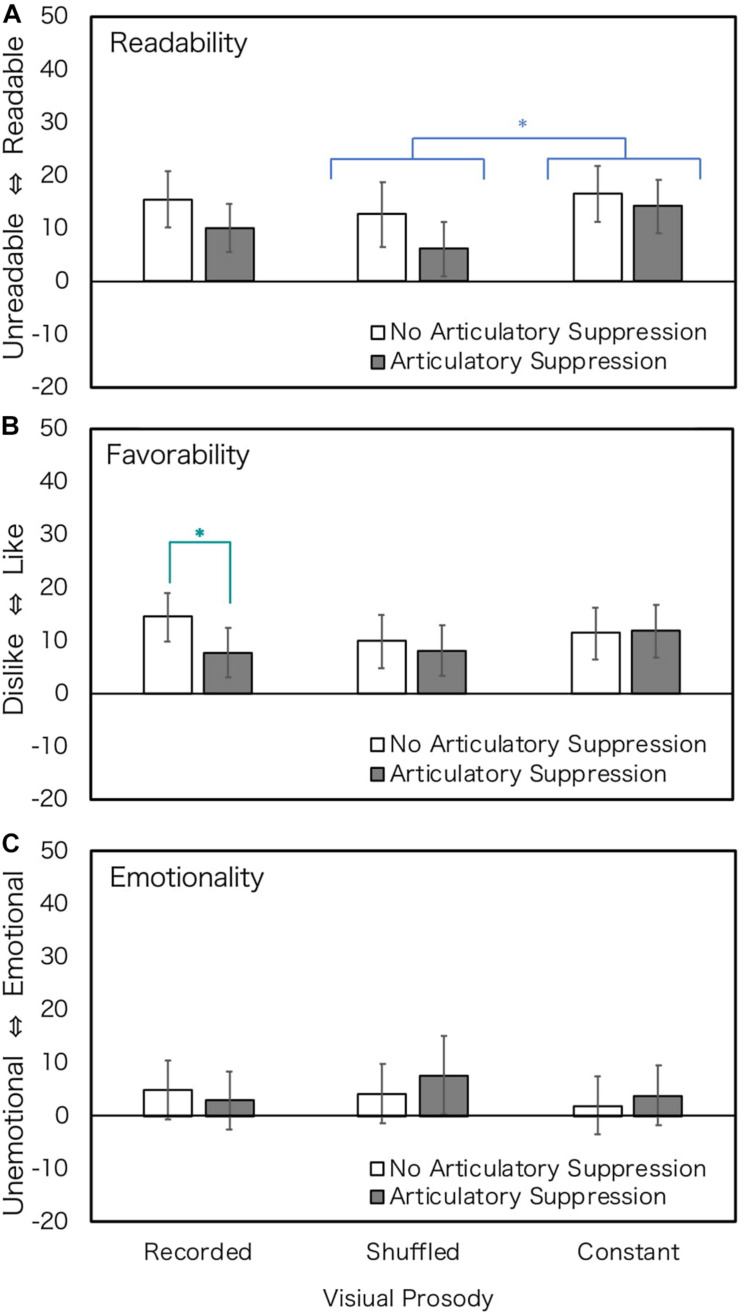
Rating values of Experiment 2 (normal hearing; all texts). Error bars show 95% confidential intervals. Data of four texts were merged. Blue stars show the effect of prosody, and a green bold star show the effect of articulatory suppression. ^∗^*p* < 0.05.

The results are shown in Figure 3. The results of ANOVA showed that the main effect of articulatory suppression was not observed in any of the impressions, and the main effect of prosody type was observed only in readability judgment, although the effect size was not large. The Bonferroni-corrected main effects revealed that the scores of constant prosody were higher than those of shuffled prosody in readability. The interaction of articulatory suppression and prosody type was significant only for favorability judgment, suggesting that the rating of recorded prosody under the articulatory suppression condition was lower than that in the non-suppression condition in favorability. The differences between the prosodies were not so clear. Table 2 summarizes the results.

**TABLE 2:** Results of ANOVA of impression of reading in Experiment 2 (normal hearing; all texts).

Impression	Effect	*F*-value, *p-*value, η*_*p*_*^2^ of main effects and interactions	Multiple comparison (bonferroni)
Readability	Articulatory suppression	*F*(1, 43) = 4.02, *p* = 0.051, η*_*p*_*^2^ = 0.09	
	Prosody type	*F (2, 86) = 5.26, *p* = 0.007, η*_*p*_*^2^ = 0.11	Shuffled < Constant
	Articulatory suppression × prosody type	*F* (1.61, 69.34) = 0.92, *p* = 0.385, η*_*p*_*^2^ = 0.02	
Favorability	Articulatory suppression	*F* (1, 43) = 2.32, *p* = 0.135, η*_*p*_*^2^ = 0.05	
	Prosody type	*F* (2, 86) = 1.54, *p* = 0.220, η*_*p*_*^2^ = 0.04	
	Articulatory suppression × prosody type	*F (1.75, 75.09) = 3.35, *p* = 0.046, η*_*p*_*^2^ = 0.07	Recorded: Articulatory suppression < No articulatory suppression
Emotionality	Articulatory suppression	*F* (1, 43) = 0.31, *p* = 0.579, η*_*p*_*^2^ = 0.01	
	Prosody type	*F* (2, 86) = 1.68, *p* = 0.193, η*_*p*_*^2^ = 0.04	
	Articulatory suppression × prosody type	*F* (1.39, 59.75) = 1.39, *p* = 0.252, η*_*p*_*^2^ = 0.03	

*p < 0.05.

### Discussion

Results showed that impressions of readability and favorability tended to be worse under articulatory suppression condition. But the effect of articulatory suppression was significant only for recorded prosody in favorability judgment. We predicted that reading texts with textual prosody requires phonological coding if the suppression impairs the impression of reading at least one condition, and that only recorded prosody would be affected by articulatory suppression. Readers with normal hearing had some difficulties storing the information of recorded prosody in the articulatory suppression condition, and as a result, the impression of the prosody worsened. This indicates that a part of textual prosody, although it is visual, is stored in the articulatory loop. The effect, however, was marginal.

Although the articulatory non-suppression condition in this experiment is exactly that of the 6 LPS condition in Experiment 1, the rating values were lower, and the effect of prosody type was smaller in Experiment 2. The reason for these results may be that Experiment 2 focused only on the optimum speed, that is, 6 LPS. Participants might rate their impressions as higher at the optimum speed, as compared to the less optimum speeds in Experiment 1. On the other hand, the rating values in Experiment 2 were not very high at the optimum speed. This may be because the participants were unable to compare their impressions to the less optimum speed and a floor effect may have occurred. Thus, we could hardly observe the effect of prosody (except for the effect of shuffled and constant prosody in readability) in this experiment.

## Experiment 3

Experiment 1 indicated that textual prosody could enrich impressions of dynamic texts in readers with normal hearing. This finding raises the question of whether textual prosody could enrich impressions of reading in people with hearing loss. Some previous studies investigating speech production found that prosodies of people with hearing loss are different from those of people with normal hearing (e.g., Stathopoulos et al., 1986; Lenden and Flipsen, 2007). However, Hanson and Fowler (1987) suggested that readers with hearing loss could access phonological information. While the effect of textual prosody might vary depending on acoustic experiences (cf. Connor and Zwolan, 2004; Sainz and de la Torre, 2005; Gallego et al., 2016), the effect might be smaller in readers with hearing loss than in readers with normal hearing. Moreover, Experiment 2 showed that a part of textual prosody was stored auditorily although it was visual in nature. Thus, for readers with hearing loss, it is predicted that the effects of textual prosody are small. However, textual prosody could enrich impressions of texts in people with hearing loss if they apply prosody processing used in sign language or lip reading to textual prosody processing. In this experiment, we examined the effects of textual prosody and presentation speed with readers with hearing loss.

### Materials and Methods

#### Participants

Twenty-six (10 males and 16 females) people with hearing loss participated in this experiment. The sample size was determined based on Experiment 1. Their mean age was 23.36 years (SD: 3.40), and their mean schooling length was 15.04 years (SD: 1.32).

All participants had physical disability certificates and relatively severe hearing loss. Self-reported impairment types were as follows; 23 were sensorineural, 1 was conductive, 1 was neurogenic, and 1 was unclear. Twenty-two of the participants had hearing loss from birth; out of these, four had hearing loss that subsequently worsened. The hearing capacities of both ears were as follows: 22 participants were at ≥ 100 dB, 2 were at ≥ 90 dB, 1 was at ≥ 80 dB, and 1 was at ≥ 70 dB.

Information about their communication methods was obtained using a multiple-choice question; 100% used sign language, 88.5% used lip reading, 82.7% used spoken language, 69.2% used writing, and 69.2% used acoustic aids. They were also asked to rate their sign language and written communication skills on a four-point scale with the following options: “very good,” “good,” “bad,” and “very bad.” In terms of sign language, 10 reported with “very good” and 16 with “good.” On written communication, 8 reported with “very good,” 13 with “good,” and 4 with “bad.”

The participants were paid ¥5,000 (roughly $50). All participants rated the texts “Thank you” and “Telegram,” while only seventeen [5 males and 12 females; mean 23.29 years (SD: 3.53)] rated “Weather forecast” and “Earthquake warning” because of sufficient time to read four texts. This experiment was performed in compliance with the Declaration of Helsinki. The protocol was approved by the Ethics Committee of Hakodate Junior College (approval number: H21-02). This study was carried out in accordance with the recommendations of Provisions of Experiments, Ethics Committee of Hakodate Junior College with written informed consent from all subjects.

#### Variables of Stimuli, Text Stimuli, and Procedures

The variables, text stimuli, and procedures were the same as in Experiment 1. Note that participants were divided into three groups for “Thank you” and “Telegram” and into two groups for “Weather forecast” and “Earthquake warning.” The order of trials for each text was randomized and different for each group.

### Results

We merged the values of the four texts because the tendencies of data in the four texts were similar. A two-way within-subject ANOVA, with prosody type and presentation speed as factors and the rating value as the dependent value, was conducted for each judgment. The degree of freedom was corrected by Greenhouse-Geisser correction when the Mauchly’s sphericity test was found to be significant. When the main effect was significant, we tested it using Bonferroni corrections.

**FIGURE 4:**
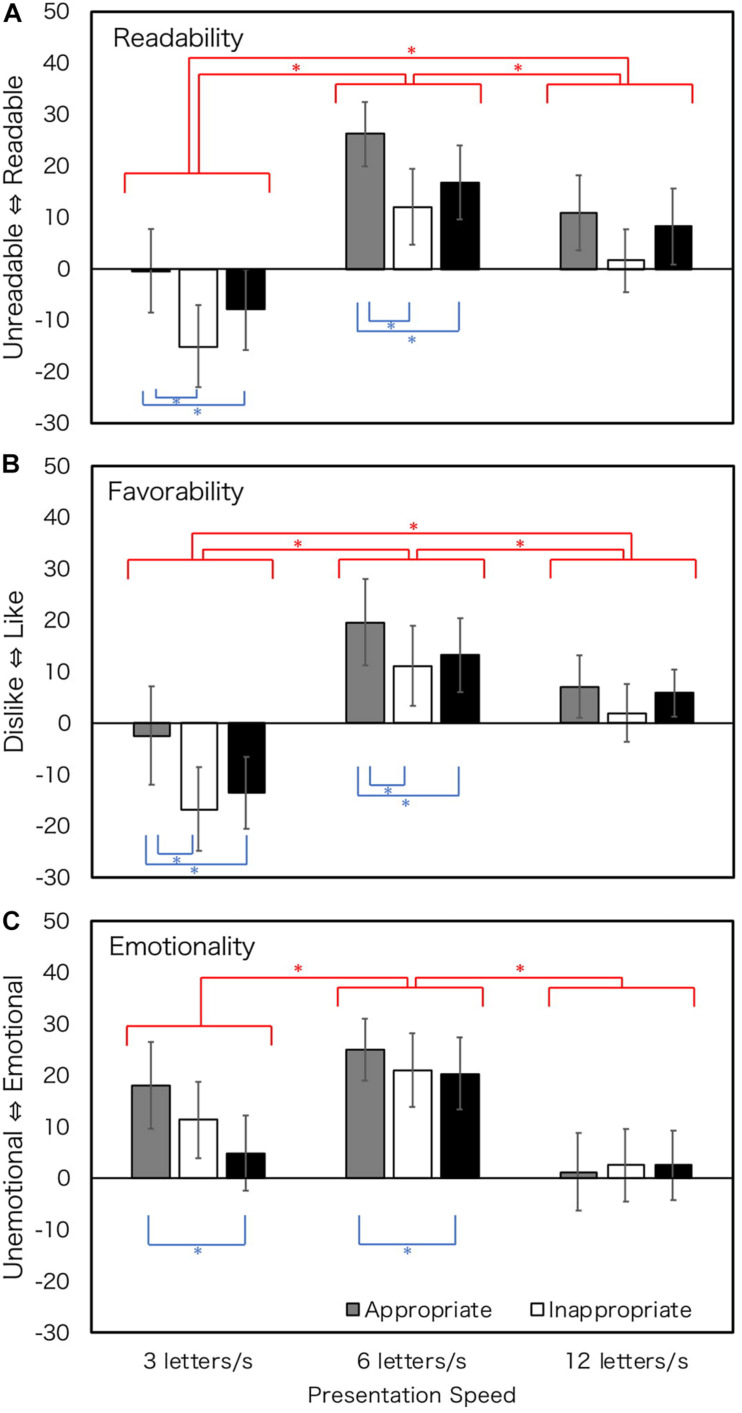
Rating values of Experiment 3 (hearing loss; all texts). Error bars show 95% confidential intervals. Data of four texts were merged. Blue stars show the effect of prosody, and red stars show the effect of presentation speed. ^∗^*p* < 0.05.

The results are shown in Figure 4. The results of ANOVA showed that the main effects of speed and the main effects of prosody type were observed in all judgments. However, the interactions of speed and prosody type were not significant for all judgments. Table 3 summarizes the results. In terms of presentation speed, Bonferroni-corrected main effect revealed that 6 LPS tended to have more readable, favorable, and emotional impressions, irrespective of prosody types. For textual prosody, recorded prosody tended to have more positive impressions than shuffled or constant prosody at 3 and 6 LPS for all judgments, but this tendency was not clear at 12 LPS.

**TABLE 3:** Results of ANOVA of impression of reading in Experiment 3 (hearing loss; all texts).

Impression	Effect	*F* value, *p* value, η*_*p*_*^2^ of main effects and interactions	Multiple comparison (bonferroni)
Readability	Speed	*F(2, 50) = 31.37, *p* < 0.0001, η*_*p*_*^2^ = 0.56	3 < 12 < 6 LPS
	Prosody type	*F(2, 50) = 13.63, *p* < 0.0001, η*_*p*_*^2^ = 0.35	Shuffled, Constant < Recorded
	Speed × prosody type	*F*(4, 100) = 0.59, *p* = 0.671, η*_*p*_*^2^ = 0.02	
Favorability	Speed	*F(2, 50) = 33.84, *p* < 0.0001, η*_*p*_*^2^ = 0.58	3 < 12 < 6 LPS
	Prosody type	*F(2, 50) = 8.67, *p* = 0.001, η*_*p*_*^2^ = 0.26	Shuffled, Constant < Recorded
	Speed × prosody type	*F*(4, 100) = 1.33, *p* = 0.264, η*_*p*_*^2^ = 0.05	
Emotionality	Speed	*F(2, 50) = 20.47, *p* < 0.0001, η*_*p*_*^2^ = 0.45	12 < 3 < 6 LPS
	Prosody type	*F(2, 50) = 3.29, *p* = 0.046, η*_*p*_*^2^ = 0.12	Constant < Recorded
	Speed × prosody type	*F*(2.95, 73.70) = 2.02, *p* = 0.120, η*_*p*_*^2^ = 0.08	

*p < 0.05.

#### Effects of Nature of Impairment

**FIGURE 5:**
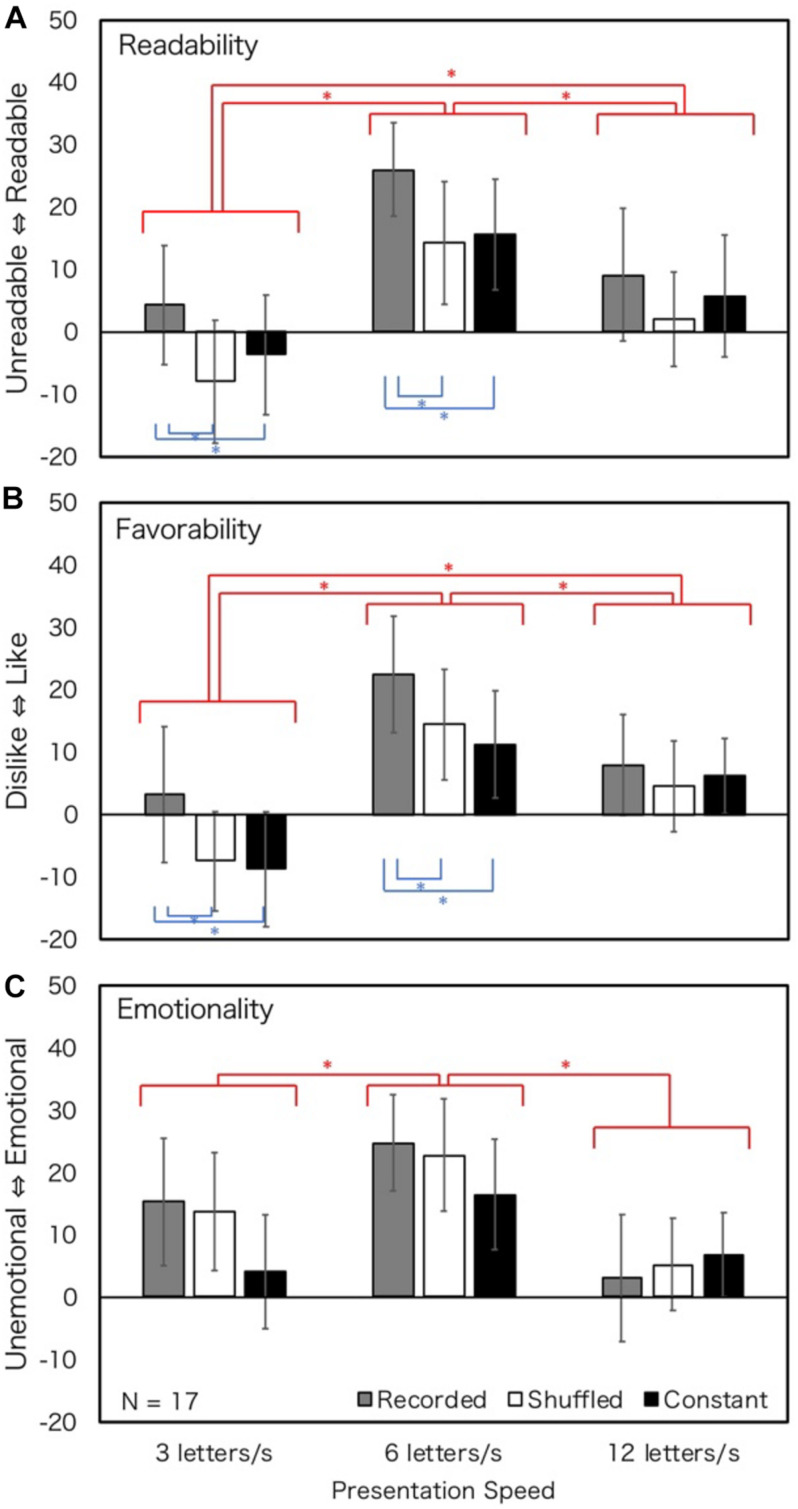
Rating values of Experiment 3 (sensorineural hearing loss from birth; all texts). Error bars show 95% confidential intervals. Data of four texts were merged. Blue stars show the effect of prosody, and red stars show the effect of presentation speed. ^∗^*p* < 0.05.

**FIGURE 6:**
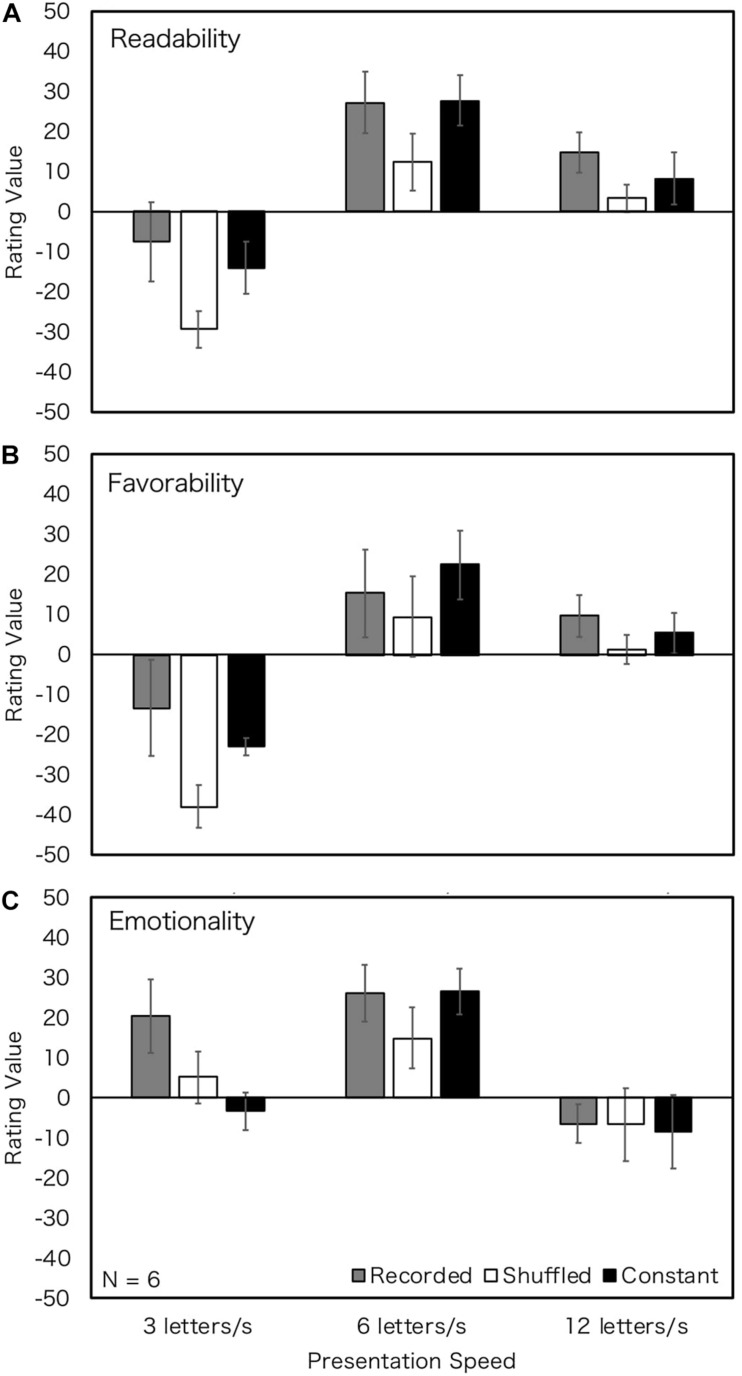
Rating values of Experiment 3 (sensorineural hearing loss not from birth; all texts). Error bars show 95% confidential intervals. Data of four texts were merged.

Performances of people with hearing loss might depend on their nature of hearing impairment. Most participants in this study had sensorineural hearing impairment. The roots of sensorineural hearing loss are located in the inner ear, the vestibulocochlear nerve, or the central auditory processing center (Koylu et al., 2016; Wang et al., 2016). Among reported hearing loss cases, 90% are cases of sensorineural hearing loss (Wang et al., 2016). People with sensorineural hearing loss are different from those with normal hearing in terms of brain structure (Wang et al., 2016). The cortical activation patterns of profound sensorineural hearing loss have revealed that the primary auditory cortex does not respond to sound stimulation in such cases (Lee et al., 2004). Lapointe et al. (2006) noted that a part of sensorineural hearing loss shows migrational abnormalities of the central nervous system. Regarding the cognitive ability of hearing loss, the lack of hearing experience in early postnatal life affects language skills (Kuhl et al., 2005) and working memory (Burkholder and Pisoni, 2003). Based on these findings, a part of language processing may be different between people with normal hearing and hearing loss. Moreover, people with hearing loss from birth generally use sign language as their first language (L1). We presented textual prosody in written language in this study. Written language corresponding to spoken language may be their second language (L2) and it may be more difficult than L1. It is expected that people with sensorineural healing loss from birth, in particular, would have difficulties in processing or storing textual prosody. Thus, we marshaled data about sensorineural hearing impairment by birth (Figure 5) and about sensorineural hearing loss not by birth, that is, late-deafened or worsening the sensorineural hearing impairment at birth (Figure 6). Seventeen participants who were sensorineural hearing loss from birth rated “Thank you” and “Telegram,” and 11 of them rated “Weather forecast” and “Earthquake warning.” Six participants who were sensorineural hearing loss not at birth rated “Thank you” and “Telegram,” and 4 of them rated “Weather forecast” and “Earthquake warning.” We merged the values of the four texts because the tendencies of data in the four texts were similar.

A two-way within-subject ANOVA was conducted for participants with sensorineural hearing loss from birth for each judgment. The degree of freedom was corrected by Greenhouse-Geisser correction when the Mauchly’s sphericity test was found to be significant. When the main effect was significant, we tested it using Bonferroni corrections. The main effects of speed and the main effects of prosody type were observed in all judgments. However, the interactions of speed and prosody type were not significant for all judgments. Table 4 shows summarizes the results. Bonferroni-corrected main effects revealed that 6 LPS gave a more positive impression for all judgments, that is, 6 LPS tended to be more readable, favorable, and emotional, irrespective of prosody types. For textual prosody, recorded prosody was more readable and favorable at 3 and 6 LPS, but this tendency was not clear at 12 LPS and in terms of emotionality. The results of participants with sensorineural hearing loss from birth were consistent with those of all participants with haring loss.

**TABLE 4:** Results of ANOVA of impression of reading in Experiment 3 (sensorineural hearing loss from birth; all texts).

Impression	Effect	*F* value, *p* value, η*_*p*_*^2^ of main effects and interactions	Multiple comparison (bonferroni)
Readability	Speed	*F(2, 32) = 24.93, *p* < 0.0001, η*_*p*_*^2^ = 0.61	3 < 12 < 6 LPS
	Prosody type	*F(2, 32) = 16.57, *p* < 0.0001, η*_*p*_*^2^ = 0.51	Shuffled, Constant < Recorded
	Speed × Prosody type	*F*(4, 64) = 0.61, *p* = 0.660, η*_*p*_*^2^ = 0.04	
Favorability	Speed	*F(2, 32) = 26.80, *p* < 0.0001, η*_*p*_*^2^ = 0.63	3 < 12 < 6 LPS
	Prosody type	*F(2, 32) = 7.61, *p* = 0.002, η*_*p*_*^2^ = 0.32	Shuffled, Constant < Recorded
	Speed × Prosody type	*F*(4, 64) = 0.78, *p* = 0.542, η*_*p*_*^2^ = 0.05	
Emotionality	Speed	*F(2, 32) = 18.88, *p* < 0.0001, η*_*p*_*^2^ = 0.54	3, 12 < 6 LPS
	Prosody type	*F(2, 32) = 3.71, *p* = 0.036, η*_*p*_*^2^ = 0.19	-
	Speed × prosody type	*F*(2.47, 39.51) = 1.731, *p* = 0.184, η*_*p*_*^2^ = 0.10	

*p < 0.05.

We did not conduct ANOVA for participants who were sensorineural hearing loss not by birth, because of their small number. However, 6 LPS tended to be preferred, and recorded and constant prosody tended to have more positive impressions for readability and favorability. Both recorded and constant prosodies were more positive at 6 LPS, and the difference between prosodies was not clear.

### Discussion

As for the effect of presentation speed, readers with hearing loss reported the highest evaluations at 6 LPS (Figures 4–6). The results were similar for the participants with normal hearing (Figure 2), suggesting that the optimum reading speed of people with hearing loss is the same as that of people with normal hearing.

Concerning the effect of textual prosody, the full group of with hearing loss (Figure 4) and the subgroup with sensorineural hearing loss from birth (Figure 5) showed more significant advantages in recorded prosody than in constant and shuffled prosodies. The results of all the participants with hearing loss could be inferred from the sub-group with sensorineural hearing loss from birth because more than half of the participants had sensorineural hearing loss from birth. The readers with sensorineural hearing loss not at birth had high rating values in recorded prosody and constant prosodies at 6 LPS. It is suggested that people with hearing loss occurring later in life could construct their own prosody just like people with normal hearing do when constant prosody is presented.

These results suggest that textual prosody affects both readers with normal hearing and those with hearing loss. It is also suggested that, contrary to our prediction, readers with hearing loss from birth are sensitive to textual prosody (the effect sizes (η*_*p*_*^2^) were larger than those in Experiment 1) and the advantage of recorded prosody was observed. It is assumed that people with hearing loss from birth would have more experience of visual languages (i.e., sign language, lip reading, and closed caption) and would utilize it to process textual prosody. Further, it is assumed that visual information is more important for people with hearing loss from birth because they cannot rely on auditory information. Thus, they may extract more information from the visual language.

## General Discussion

In this study, we demonstrated that textual prosody and presentation speed can influence readers’ impressions. In terms of presentation speed, this study demonstrated that readers with normal hearing and hearing loss had high impression scores at 6 LPS. This speed was consistent with the optimum speed reported in previous studies (Price et al., 1996; Uetsuki et al., 2017). The results suggest that the optimum reading speed of people with hearing loss is equivalent to that of people with normal hearing.

Textual prosody was presented visually in our study, but it could convey temporal information like prosody. Recorded prosody, in which the letters appear with various intervals according to the syllables or larger unit of texts, tends to give a more positive impression than shuffled prosody at 3 and 6 LPS consistently, but the difference is not necessarily statistically significant. This tendency was confirmed both with participants with normal hearing and with those with hearing loss. This suggests that appropriate textual prosody can enrich our impression of reading below the optimum speed regardless of whether or not readers have hearing loss.

On the other hand, the effect of textual prosody almost disappears at a high presentation speed (12 LPS) for both readers with normal hearing and hearing loss. There are three possible explanations for this. First, the effect of textual prosody was large at low presentation speeds because the differences in prosody types are relatively large at lower speeds than at higher speeds. Second, 12 LPS was too fast for readers to integrate the prosodic cues. Third, recorded prosody could support sentence processing when the processing of visually presented text is difficult because of slowness in presentation speed (i.e., 3 LPS).

When texts were presented at 6 LPS, readers with sensorineural hearing loss from birth had more positive impressions in recorded prosody, although readers with normal hearing had positive impressions in both recorded and constant prosodies. There are at least three possible reasons. First, when texts have no prosody (constant prosody), it appears that normal hearing readers could construct appropriate prosodies and prefer them. Readers with sensorineural hearing loss from birth, however, could not construct appropriate prosodies by themselves. As a result, they depended on textual prosody information (recorded prosody) and have positive impressions with it than constant prosody. Second, more positive impressions of recorded prosody in readers with hearing loss could also be due to more experience with visual language (i.e., sign language, lip reading, and closed captioning). They could apply this experience to process textual prosody. Third, the recorded prosody in this study was based on the reading of only one person. Thus, the recorded prosody was not always favored because the timing or the speed may be idiosyncratic to this particular individual.

Experiment 2 showed that articulatory suppression could affect the impression of recorded prosody. It indicated that recorded prosody should be stored in the articulatory loop and that prosody information may be converted to auditory representation even when the prosody is visual. The results also indicate that the temporal structure of texts is processed regardless of whether the input is visual or auditory. Under these conditions, readers with hearing loss also might use the articulatory loop or might retain their information by using different means to store the textual prosody information. Further study is required to address these possibilities.

In sum, the results of this study indicate that the temporal information is processed regardless of whether the input is visual or auditory. The results also demonstrated that textual prosody could enrich the reading not only of people with normal hearing but also of those with hearing loss, regardless of acoustic experiences. Although people with hearing loss have less acoustic experience than people with normal hearing, they may be able to utilize their experience of visual language, such as sign language, lip reading, and closed caption for processing textual prosody. Furthermore, textual prosody could be a useful tool to convey speech speeds and timing information visually.

We should note that the enrichment of impressions was moderate in size of effect and unstable among reading contents and conditions, especially in readers with normal hearing. However, for recorded prosody, the degradation of impressions at 3 LPS could be somehow alleviated. The ratings of recorded prosody at slow speed were not as high as those of constant prosody at 6 LPS. Recorded prosody may support text processing when the processing does not go smoothly by giving readers clues to the structures of sentence. For example, multimedia situations where texts, music, and/or images are presented simultaneously (e.g., role-playing games, TV programs, and movies) should be difficult for readers to concentrate text processing. Textual prosody, however, may engage readers’ attention and enrich text impressions under the multimedia situations. Textual prosody can achieve novel and people-friendly impressions of reading in digital books, TV or films.

We have demonstrated the effectiveness of textual prosody (varying the speed and the timings of pause) in digital text presentation. However, there are also other types of dynamic text presentation. For example, scroll display, in which texts move from left to right, is often used on TV news or electronic message boards. It is difficult for scroll display to convey prosodic information, resulting in visual processing difficulties such as visual masking (Breitmeyer and Ogmen, 2000) and motion blur (Wong and Halvorsen, 2006). On the other hand, the method used in this study can avoid these visual processing difficulties because the letters do not move.

Some studies have tried to express prosodic information with written text. For example, Patel and McNab (2011) used relatively limited participants (children ages 6–9 years old) and examined whether explicit visual cues of the target prosody would facilitate appropriate modulation of these cues when children read aloud. They provided temporal changes in pitch and duration as the change in spatial position of characters (for example, spacing between characters to indicate word duration, spacing between words to signal pause duration, and fitting characters to the F0 contour of the adult model’s productions to indicate F0 variation). These textual manipulations conveying prosody can improve children’s reading expressivity. However, in their method, the readability decreased because the letters were not level. In contrast, our method can avoid this issue because temporal changes of prosody are expressed as temporal changes without spatial position shifts.

Some issues must be considered. First, our discussion is based merged results based on four text stimuli. In the Supplementary Material, we show the results for the four text types and three impression types separately, and it suggests the possibility that the effects of textual prosody vary slightly according to text types. The effect of textual prosody was not evident depending on text types because the effect of textual prosody was not strong, though the effect should be apparent when large numbers of readers participate. Second, the number of participants with hearing loss was fairly small except for that of sensorineural hearing loss from birth. It is not clear whether our findings are valid for other hearing loss. This calls for more detailed data with other hearing loss.

## Data Availability Statement

All datasets presented in this study are included in the article/Supplementary Material.

## Ethics Statement

The studies involving human participants were reviewed and approved by the Ethics Committee of Hakodate Junior College and Ethics committee of Aoyama Gakuin University. The patients/participants provided their written informed consent to participate in this study.

## Author Contributions

MU, KM, and JW conceived, designed the experiments and wrote up the study. MU performed the experiments and analyzed the data. KM and JW contributed the software. All authors contributed to the article and approved the submitted version.

## Conflict of Interest

KM and JW were employed by Communication Science Laboratories, Nippon Telegraph and Telephone Corporation. The remaining author declares that the research was conducted in the absence of any commercial or financial relationships that could be construed as a potential conflict of interest.

## Appendix

Table A1 shows Japanese text stimuli used in this paper.

**TABLE A1:** Text types and its Japanese text stimuli

Text type	Letters of text	Morae of text	Japanese text	English translation
Thank you	17	16		Thank you very much all the time.
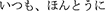
			[itsumo, hontou-ni	

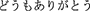
				
			doumo arigatou.]	
Telegram	40	46		You kept at a long and arduous task, and you achieved success. Now you are in a good spring where many flowers bloom. Congratulations for passing the exam.
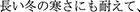
			[nagai fuyu-no samusa nimo tae-te,	

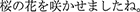
				
			sakura-no hana-o sakase mashitane.	

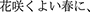
				
			hana saku yoi haru-ni,	

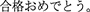
				
			goukaku omedetou.]	
Weather forecast	55	63		Same as yesterday, the area around Japan is in a winter-style air pressure arrangement. It is cloudy in the central city of Tokyo today, and it will rain in some places.
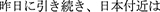
			[kinou-ni hikituduki, nihon fukin-ha	

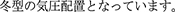
				
			fuyugata-no kiatsu haichi-to natte imasu.	

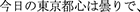
				
			kyou-no Tokyo toshin-ha kumori-de,	

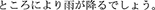
				
			tokoro-ni yori ame-ga furu de shou.]	
Earthquake Warning	22	29		This is an emergency earthquake flash report. Please beware of a strong shake.
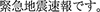
			[kinkyu jishin sokuhou desu.	

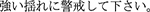
				
			tsuyoi yure-ni keikai shite kudasai.]	
