# Supplementary material for: “Textual Prosody” Can Change Impressions of Reading in People With Normal Hearing and Hearing Loss
Source: Front Psychol. 2020 Dec 17;11:548619. doi: 10.3389/fpsyg.2020.548619 (PMC7773827; doi:10.3389/fpsyg.2020.548619)
Supplement: Supplementary file 2 [file Table_1.PDF]

Supplementary Material

1 Figures and Tables That Showed in Text Types Separately

In this section, we show data in text types and impression types separately. The overall tendency of these data was not different from the merged data in the Results, and the basic effects of textual prosody and presentation speed were observed. However, the effect did not appear robust. The effects were small in each text stimuli. This may have been because participants' favorability affects rating values. When we increase the number of participants, the data should be closer to the merged data shown in the Results.

1.1 Participants with Normal Hearing

Supplementary Figs. 1-4 and Supplementary Tables 1-4 show the results of Experiment 1 for the four text types and three impression types separately. Six LPS is favored in "Thank you," "Telegram," and "Weather Forecast." However, 12 LPS is preferred only in the text of "Earthquake Warning." When "Earthquake Warning" is presented, the text must be read quickly because it is life-threatening information.

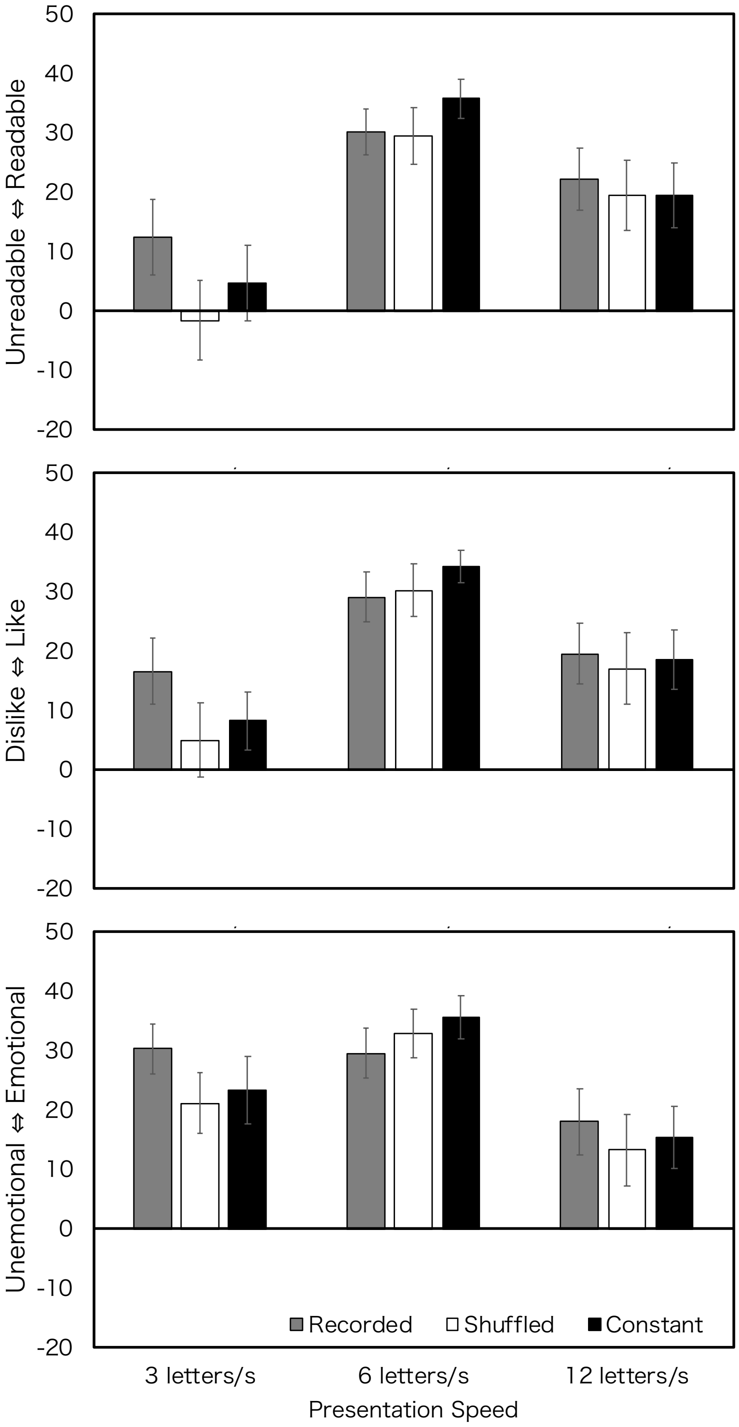

Supplementary Fig. 1. Rating values of Experiment 1 (Normal Hearing; Thank you)

Error bars show 95% confidential intervals.

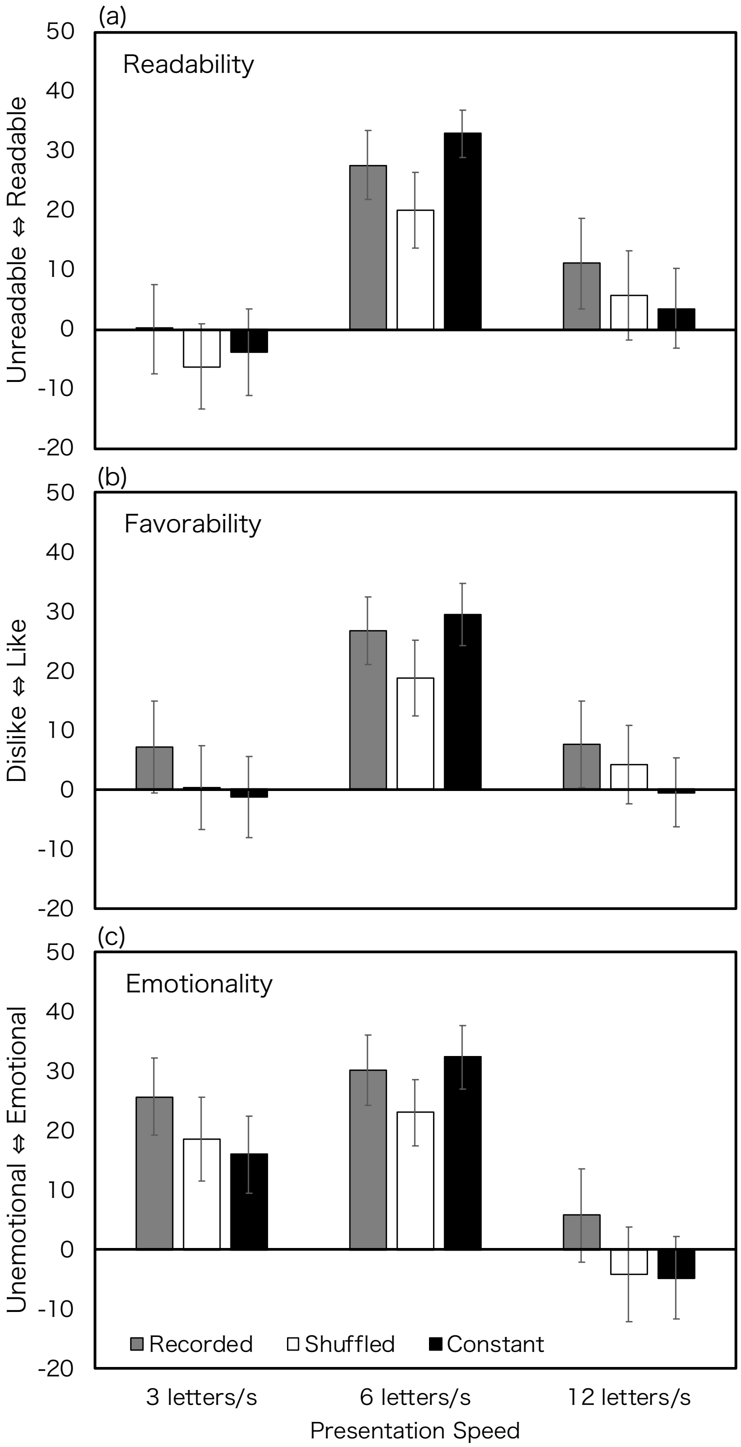

Supplementary Fig. 2. Rating values of Experiment 1 (Normal Hearing; Telegram)

Error bars show 95% confidential intervals.

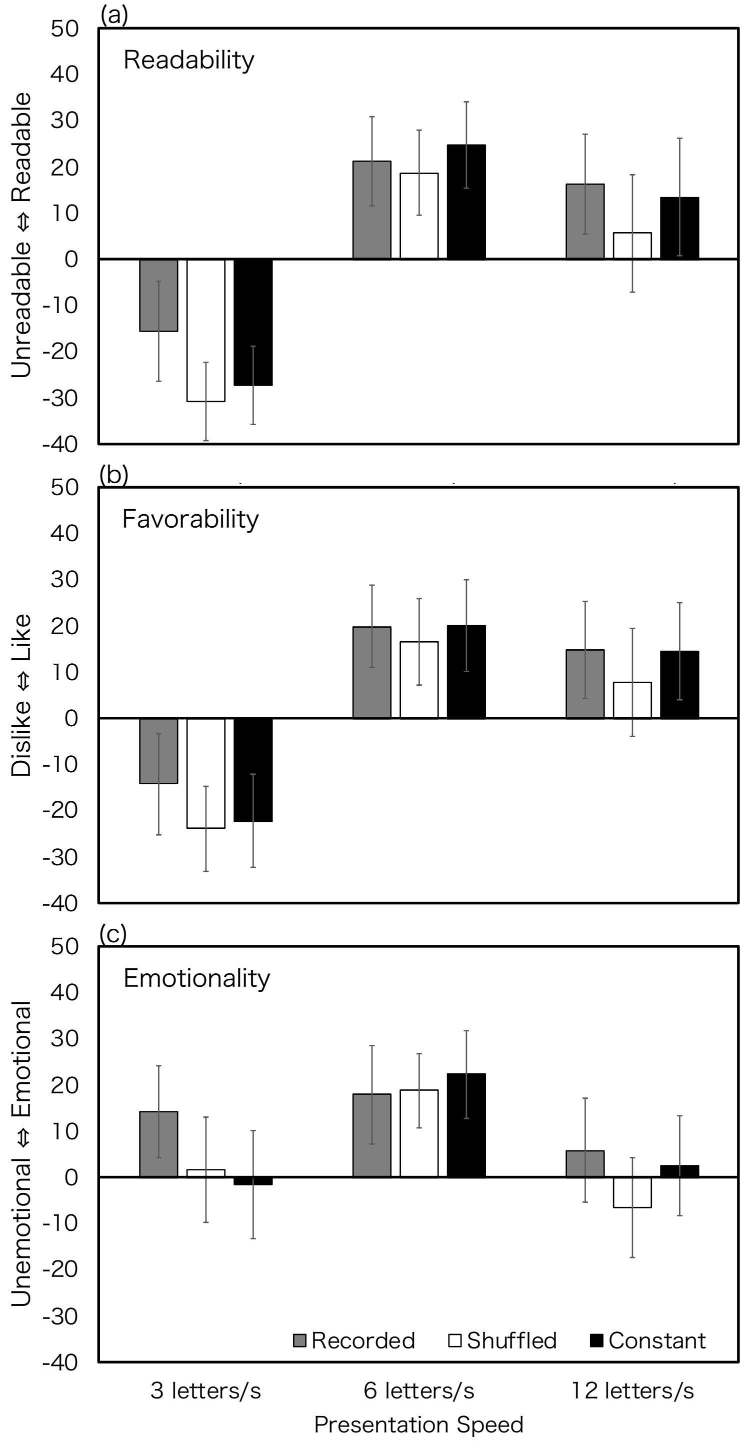

Supplementary Fig. 3. Rating values of Experiment 1 (Normal Hearing; Weather Forecast)

Error bars show 95% confidential intervals.

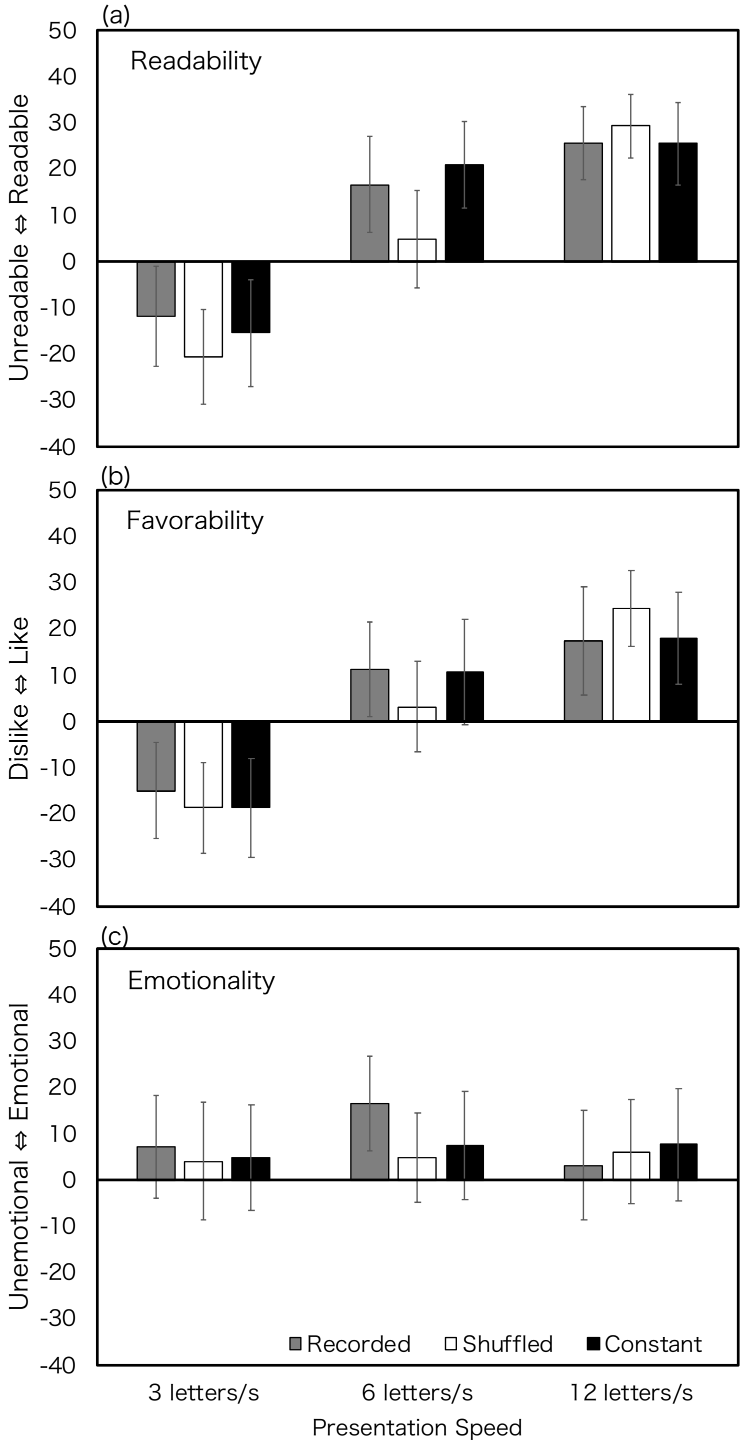

Supplementary Fig. 4. Rating values of Experiment 1 (Normal Hearing; Earthquake Warning)

Error bars show 95% confidential intervals.

Supplementary Table 1. Results of ANOVA for Impression of Reading in Experiment 1 (Normal Hearing; Thank You).

Impression	Effect	*F* value, *p* value, η_p^2 of Main Effects, Interactions, and Simple Main Effects	Multiple Comparison (Bonferroni)
Readability	Speed	$F(1.48,75.47) = 50.630$ $p < .0001$, $\eta_p^2 = .50$ ***	
	Prosody Type	$F(2,102) = 8.26$, $p < .0001$, $\eta_p^2 = .14$ ***	
	Speed × Prosody Type	$F(3.26,166.04) = 5.51$, $p = .001$, $\eta_p^2 = .10$ **<br><br>Simple Main Effects<br>Presentation speed at Recorded: F(2,50) = 26.92, p < .0001, $\eta_p^2 = .52$<br>Presentation speed at Shuffled: F(2,50) = 40.10, p < .0001, $\eta_p^2 = .62$<br>Presentation speed at Constant: F(2,50) = 58.36, p < .0001, $\eta_p^2 = .70$<br><br>Prosody Type at 3 LPS: F(2,50) = 12.14, p < .0001, $\eta_p^2 = .33$<br>Prosody Type at 6 LPS: F(2,50) = 4.34, p = .018, $\eta_p^2 = .15$<br>Prosody Type at 12 LPS: F(2,50) = .73, p = .487, $\eta_p^2 = .03$	Recorded, Shuffled, Constant: 3 < 12 < 6 LPS<br><br>3 LPS: Shuffled, Constant < Recorded<br>6 LPS: Shuffled, Recorded < Constant
Favorability	Speed	$F(1.71,87.33) = 48.30$, $p < .0001$, $\eta_p^2 = .49$ ***	
	Prosody Type	$F(2,102) = 4.51$, $p = .013$, $\eta_p^2 = .08$ *	
	Speed × Prosody Type	$F(3.22,164.26) = 4.32$, $p = .005$, $\eta_p^2 = .08$ **<br><br>Simple Main Effects<br>Presentation speed at Recorded: F(2,50) = .625, p < .0001, $\eta_p^2 = .38$<br>Presentation speed at Shuffled: F(2,50) = 32.00, p < .0001, $\eta_p^2 = .56$<br>Presentation speed at Constant: F(2,50) = 54.92, p < .0001, $\eta_p^2 = .69$<br><br>Prosody Type at 3 LPS: F(2,50) = 7.57, p = .001, $\eta_p^2 = .23$<br>Prosody Type at 6 LPS: F(2,50) = 3.31, p = .045, $\eta_p^2 = .12$<br>Prosody Type at 12 LPS: F(2,50) = .29, p = .753, $\eta_p^2 = .01$	Recorded: 3, 12 < 6 LPS<br><br>Shuffled, Constant: 3 < 12 < 6 LPS<br><br>3 LPS: Shuffled, Constant < Recorded
Emotionality	Speed	$F(1.48,75.32) = 32.37$, $p < .0001$, $\eta_p^2 = .39$ ***	
	Prosody Type	$F(2,102) = 3.01$, $p = .054$, $\eta_p^2 = .06$	
	Speed × Prosody Type	$F(4,204) = 3.65$, $p = .007$, $\eta_p^2 = .07$ **<br><br>Simple Main Effects<br>Presentation speed at Recorded: F(2,50) = 9.61, p < .0001, $\eta_p^2 = .28$<br>Presentation speed at Shuffled: F(2,50) = 27.15 p < .0001, $\eta_p^2 = .52$<br>Presentation speed at Constant: F(2,50) = 27.56, p < .0001, $\eta_p^2 = .52$<br><br>Prosody Type at 3 LPS: F(2,50) = 7.21, p = .002, $\eta_p^2 = .22$<br>Prosody Type at 6 LPS: F(2,50) = 3.32, p = .044, $\eta_p^2 = .12$<br>Prosody Type at 12 LPS: F(2,50) = 1.19, p = .312, $\eta_p^2 = .05$	Recorded: 12 < 3, 6 LPS<br><br>Shuffled, Constant: 3, 12 < 6 LPS<br><br>3 LPS: Shuffled, Constant < Recorded<br>6 LPS: Recorded < Constant

Supplementary Table 2. Results of ANOVA for Impression of Reading in Experiment 1 (Normal Hearing; Telegram).

Impression	Effect	F value, p value, η_p^2 of Main Effects, Interactions, and Simple Main Effects	Multiple Comparison (Bonferroni)
Readability	Speed	$F(1.77,90.44) = 53.10, p < .0001, \eta_p^2 = .51$ ***	
	Prosody Type	$F(2,102) = 4.78, p = .010, \eta_p^2 = .09$ *	
	Speed × Prosody Type	$F(4,204) = 2.55, p = .041, \eta_p^2 = .05$ *<br><br>Simple Main Effects<br>Presentation speed at Recorded: F(2,50) = 21.80, p < .0001, $\eta_p^2 = .47$<br>Presentation speed at Shuffled: F(2,50) = 20.19 p < .0001, $\eta_p^2 = .45$<br>Presentation speed at Constant: F(2,50) = 54.23, p < .0001, $\eta_p^2 = .68$<br><br>Prosody Type at 3 LPS: F(2,50) = .98, p = .382, η_p^2 = .04<br>Prosody Type at 6 LPS: F(2,50) = 6.48, p = .003, η_p^2 = .21<br>Prosody Type at 12 LPS: F(2,50) = 2.00, p = .146, $\eta_p^2 = .07$	Recorded: 3 < 12 < 6 LPS<br><br>Shuffled, Constant: 3, 12 < 6 LPS<br><br>6 LPS: Shuffled < Constant
Favorability	Speed	$F(1.77,90.44) = 53.10, p < .0001, \eta_p^2 = .51$ ***	
	Prosody Type	$F(2,102) = 4.78, p = .010, \eta_p^2 = .09$ *	
	Speed × Prosody Type	$F(4,204) = 2.54, p = .041, \eta_p^2 = .05$ *<br><br>Simple Main Effects<br>Presentation speed at Recorded: F(2,50) = 14.70, p < .0001, $\eta_p^2 = .37$<br>Presentation speed at Shuffled: F(2,50) = 11.47 p < .0001, $\eta_p^2 = .31$<br>Presentation speed at Constant: F(2,50) = 40.91, p < .0001, $\eta_p^2 = .62$<br><br>Prosody Type at 3 LPS: F(2,50) = 1.74, p = .185, η_p^2 = .07<br>Prosody Type at 6 LPS: F(2,50) = 4.74, p = .013, η_p^2 = .16<br>Prosody Type at 12 LPS: F(2,50) = 1.87, p = .165, $\eta_p^2 = .07$	Recorded: 3 < 12 < 6 LPS<br><br>Shuffled, Constant: 3, 12 < 6 LPS<br><br>6 LPS: Shuffled < Constant
Emotionality	Speed	$F(1.70,8.73) = 46.919, p < .0001, \eta_p^2 = .48$ ***	12 < 3 < 6 LPS
	Prosody Type	$F(2,102) = 6.62, p = .002, \eta_p^2 = .12$ **	Shuffled < Recorded
	Speed × Prosody Type	$F(4,204) = 1.90, p = .111, \eta_p^2 = .04$	

Supplementary Table 3. Results of ANOVA for Impression of Reading in Experiment 1 (Normal Hearing; Weather Forecast).

Impression	Effect	*F* value, *p* value, η_p^2 of Main Effects, Interactions, and Simple Main Effects	Multiple Comparison (Bonferroni)
Readability	Speed	$F(2,102) = 122.36, p < .0001, \eta_p^2 = .71$ ***	
	Prosody Type	$F(2,102) = 6.52, p = .002, \eta_p^2 = .11$ **	
	Speed × Prosody Type	$F(4,204) = 2.66, p = .034, \eta_p^2 = .05$ *<br><br>Simple Main Effects<br>Presentation speed at Recorded: F(2,50) = 41.90, p < .0001, $\eta_p^2 = .62$<br>Presentation speed at Shuffled: F(2,50) = 89.58 p < .0001, $\eta_p^2 = .78$<br>Presentation speed at Constant: F(2,50) = 100.74, p < .0001, $\eta_p^2 = .80$<br><br>Prosody Type at 3 LPS: F(2,50) = 10.47, p < .0001, $\eta_p^2 = .30$<br>Prosody Type at 6 LPS: F(2,50) = 1.35, p = .169, $\eta_p^2 = .05$<br>Prosody Type at 12 LPS: F(2,50) = 2.82, p = .069, $\eta_p^2 = .10$	Recorded, Constant: 3 < 6, 12 LPS<br><br>Shuffled: 3 < 12 < 6 LPS<br><br><br>3LPS: Shuffled, Constant < Recorded
Favorability	Speed	$F(2,102) = 97.99, p < .0001, \eta_p^2 = .66$ ***	3 < 6, 12 LPS
	Prosody Type	$F(2,102) = 4.97, p = .009, \eta_p^2 = .09$ **	Shuffled < Recorded
	Speed × Prosody Type	$F(4,204) = 1.00, p = .408, \eta_p^2 = .02$	
Emotionality	Speed	$F(2,102) = 25.951\ p < .0001, \eta_p^2 = .34$ ***	
	Prosody Type	$F(2,102) = 7.02, p = .001, \eta_p^2 = .12$ **	
	Speed × Prosody Type	$F(4,204) = 4.48, p = .002, \eta_p^2 = .08$ **<br><br>Simple Main Effects<br>Presentation speed at Recorded: F(2,50) = 3.78, p = .030, $\eta_p^2 = .13$<br>Presentation speed at Shuffled: F(2,50) = 27.13p < .0001, $\eta_p^2 = .52$<br>Presentation speed at Constant: F(2,50) = 19.73, p < .0001, $\eta_p^2 = .44$<br><br>Prosody Type at 3 LPS: F(2,50) = 11.58, p < .0001, $\eta_p^2 = .32$<br>Prosody Type at 6 LPS: F(2,50) = 5.73 p = .485, $\eta_p^2 = .03$<br>Prosody Type at 12 LPS: F(2,50) = 6.01, p = .005, $\eta_p^2 = .19$	Recorded: 12 < 6 LPS<br><br>Shuffled, Constant: 3, 12 < 6 LPS<br><br>3 LPS: Shuffled, Constant < Recorded<br><br>12 LPS: Shuffled < Recorded, Constant

Supplementary Table 4. Results of ANOVA for Impression of Reading in Experiment 1 (Normal Hearing; Earthquake Warning).

Impression	Effect	*F* value, *p* value, η_p^2 of Main Effects, Interactions, and Simple Main Effects	Multiple Comparison (Bonferroni)
Readability	Speed	$F(1.643,83.782) = 139.95, p < .0001, \eta_p^2 = .73$ ***	
	Prosody Type	$F(2,102) = 5.39, p = .006, \eta_p^2 = .10$ **	
	Speed × Prosody Type	$F(3.24,165.28) = 4.550, p = .003, \eta_p^2 = .08$ **<br><br>Simple Main Effects<br>Presentation speed at Recorded: F(2,50) = 37.87, p < .0001, $\eta_p^2 = .60$<br>Presentation speed at Shuffled: F(2,50) = 99.07, p < .0001, $\eta_p^2 = .80$<br>Presentation speed at Constant: F(2,50) = 51.78, p < .0001, $\eta_p^2 = .467$<br><br>Prosody Type at 3 LPS: F(2,50) = 2.74, p = .074, $\eta_p^2 = .10$<br>Prosody Type at 6 LPS: F(2,50) = 9.35, p < .0001, $\eta_p^2 = .27$<br>Prosody Type at 12 LPS: F(2,50) = 1.34, p = .270, $\eta_p^2 = .05$	Recorded, Constant: 3 < 6, 12 LPS<br>Shuffled: 3 < 6 < 12 LPS<br><br>6 LPS: Shuffled < Recorded, Constant
Favorability	Speed	$F(1.74,88.95) = 105.56, p < .0001, \eta_p^2 = .67$ ***	
	Prosody Type	$F(2,102) = .30, p = .739, \eta_p^2 = .01$	
	Speed × Prosody Type	$F(3.36,171.12) = 2.90, p = .031, \eta_p^2 = .05$<br><br>Simple Main Effects<br>Presentation speed at Recorded: F(2,50) = 28.63, p < .0001, $\eta_p^2 = .53$<br>Presentation speed at Shuffled: F(2,50) = 60.29, p < .0001, $\eta_p^2 = .71$<br>Presentation speed at Constant: F(2,50) = 50.24, p < .0001, $\eta_p^2 = .67$<br><br>Prosody Type at 3 LPS: F(2,50) = .83, p = .442, $\eta_p^2 = .03$<br>Prosody Type at 6 LPS: F(2,50) = 2.84, p = .068, $\eta_p^2 = .10$<br>Prosody Type at 12 LPS: F(2,50) = 3.18, p = .050, $\eta_p^2 = .11$	Recorded, Constant: 3 < 6, 12 LPS<br>Shuffled: 3 < 6 < 12 LPS
Emotionality	Speed	$F(1.78,90.80) = 1.35, p = .264, \eta_p^2 = .03$	
	Prosody Type	$F(1.73,88.33) = 1.91, p = .159, \eta_p^2 = .04$	
	Speed × Prosody Type	$F(4,204) = 2.82, p = .026, \eta_p^2 = .05$ *<br><br>Simple Main Effects<br>Presentation speed at Recorded: F(2,50) = 9.64, p < .0001, $\eta_p^2 = .28$<br>Presentation speed at Shuffled: F(2,50) = .10, p = .908, $\eta_p^2 = .00$<br>Presentation speed at Constant: F(2,50) = .25, p = .778, $\eta_p^2 = .01$<br><br>Prosody Type at 3 LPS: F(2,50) = .41, p = .667, $\eta_p^2 = .02$<br>Prosody Type at 6 LPS: F(2,50) = 8.93, p < .0001, $\eta_p^2 = .26$<br>Prosody Type at 12 LPS: F(2,50) = .77, p = .470, $\eta_p^2 = .03$	Recorded: 3, 12 < 6 LPS<br><br>6 LPS: Shuffled < Recorded

1.2 Participants with Hearing Loss

Supplementary Figs. 5-8 and Supplementary Tables 5-8 show the results of Experiment 3 for the four text types and three impression types separately.

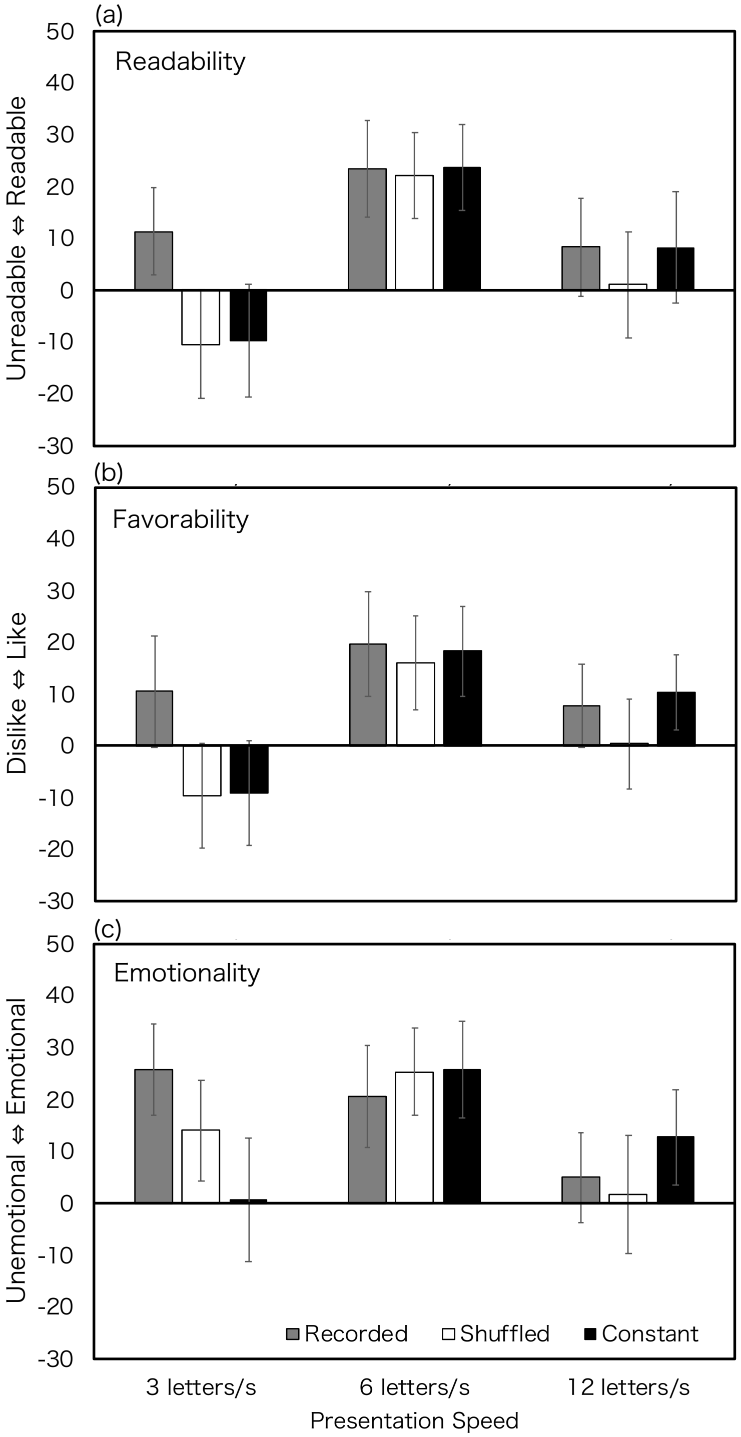

Supplementary Fig. 5. Rating values of Experiment 3 (Hearing Loss; Thank you)

Error bars show 95% confidential intervals.

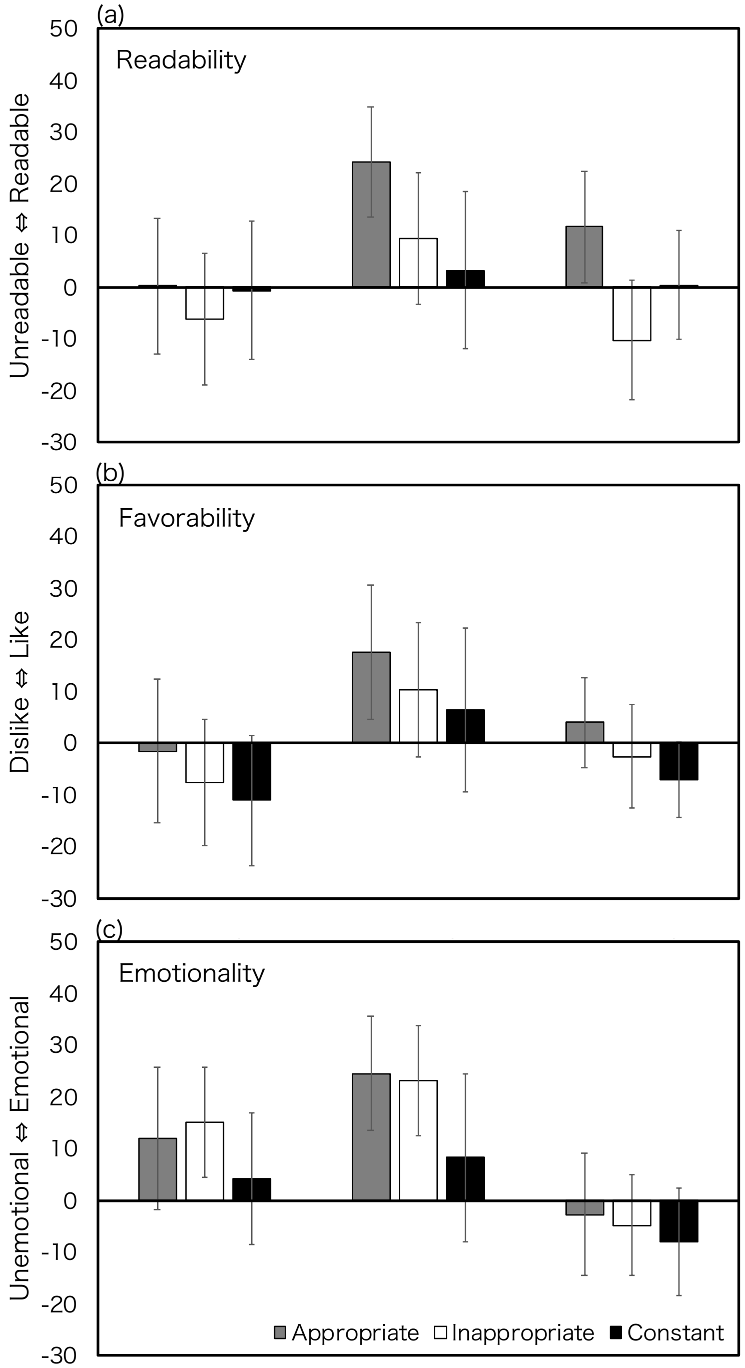

Supplementary Fig. 6. Rating values of Experiment 3 (Hearing Loss; Telegram)

Error bars show 95% confidential intervals.

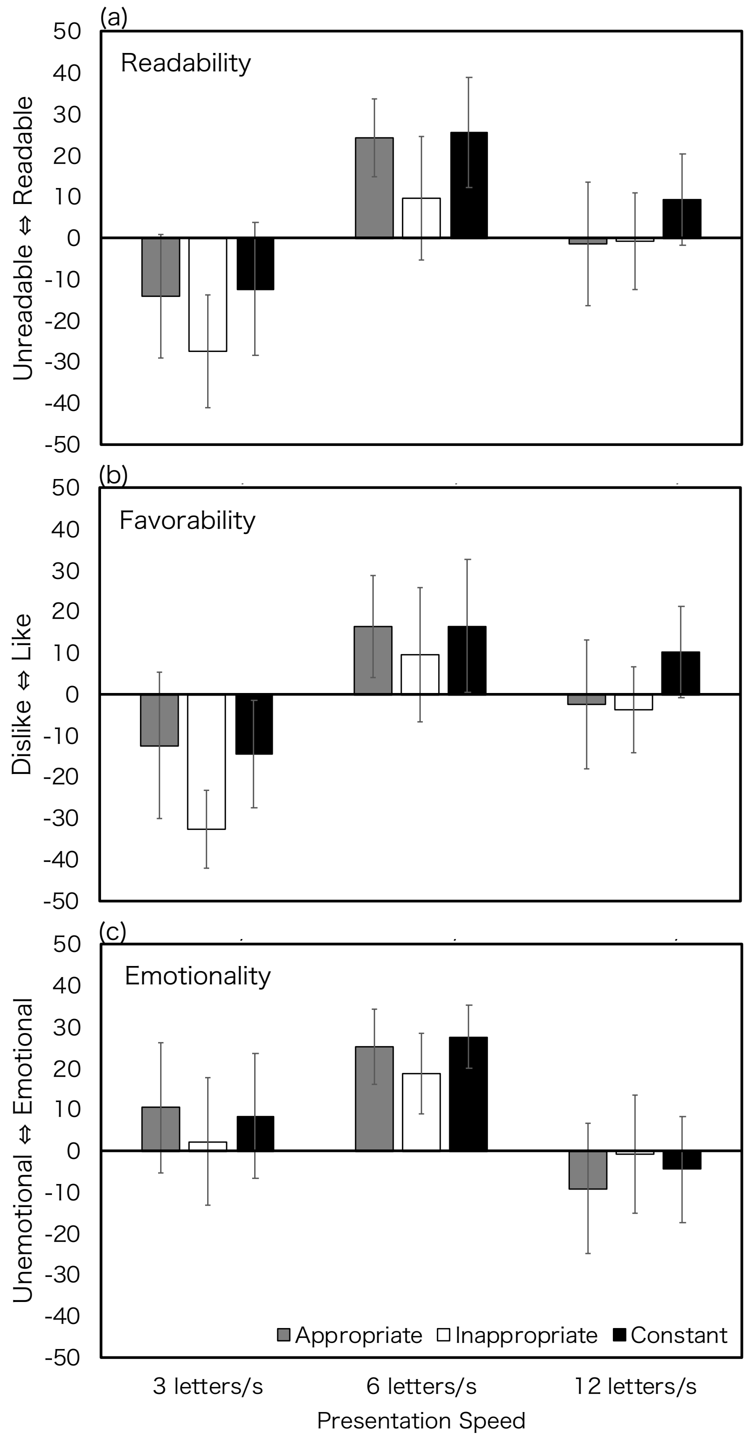

Supplementary Fig. 7. Rating values of Experiment 3 (Hearing Loss; Weather Forecast)

Error bars show 95% confidential intervals.

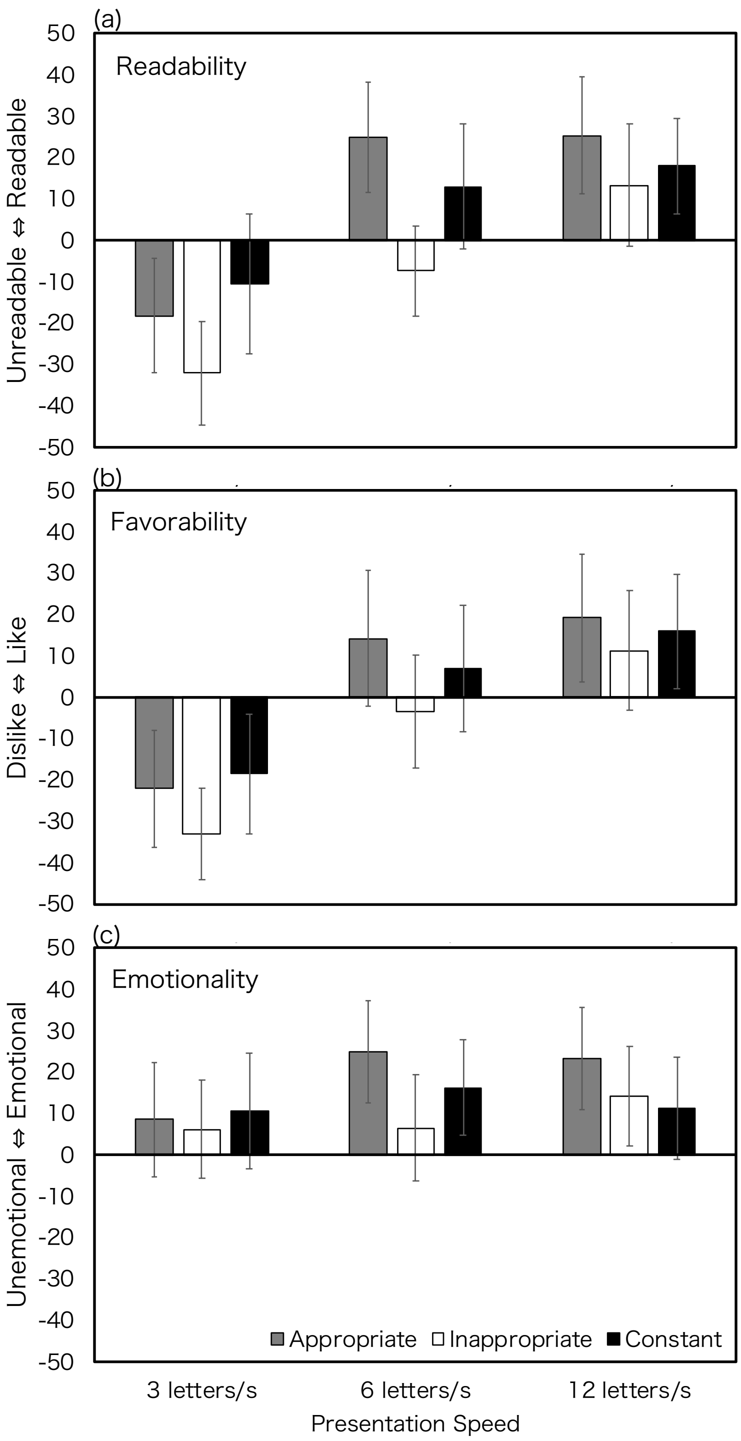

Supplementary Fig. 8. Rating values of Experiment 3 (Hearing Loss; Earthquake Warning)

Error bars show 95% confidential intervals.

Supplementary Table 5. Results of ANOVA for Impression of Reading in Experiment 3 (Hearing loss; Thank you).

Impression	Effect	F value, p value, η_p^2 of Main Effects, Interactions, and Simple Main Effects		Multiple Comparison (Bonferroni)
Readability	Speed	$F(2,50) = 17.44, p < .0001, \eta_p^2 = .41$	***	3, 12 < 6 LPS
	Prosody Type	$F(2,50) = 5.15, p = .009, \eta_p^2 = .17$	**	Shuffled < Recorded
	Speed × Prosody Type	$F(4,100) = 3.59, p = .009, \eta_p^2 = .13$<br><br>Simple Main Effects<br>Presentation speed at Recorded: F(2,24) = 4.14, p = .029, η_p^2 = .26<br>Presentation speed at Shuffled: F(2,24) = 12.29, p < .0001, η_p^2 = .51<br>Presentation speed at Constant: F(2,24) = 12.89, p < .0001, η_p^2 = .52<br><br>Prosody Type at 3 LPS: F(2,24) = 6.92, p = .004, η_p^2 = .37<br>Prosody Type at 6 LPS: F(2,24) = .11, p = .899, η_p^2 = .01<br>Prosody Type at 12 LPS: F(2,24) = .80, p = .461, η_p^2 = .06	**	Recorded:-<br><br>Shuffled: 3, 12 < 6 LPS<br><br>Constant: 3 < 6, 12 LPS<br><br>3 LPS: Shuffled, Constant < Recorded
Favorability	Speed	$F(2,50) = 12.17, p < .0001, \eta_p^2 = .33$	***	3, 12 < 6 LPS
	Prosody Type	$F(2,50) = 7.68, p = .001, \eta_p^2 = .24$	**	Shuffled < Recorded
	Speed × Prosody Type	$F(2.75,68.85) = 3.583, p = .009, \eta_p^2 = .13$<br><br>Simple Main Effects<br>Presentation speed at Recorded: F(2,24) = 3.40, p = .050, η_p^2 = .22<br>Presentation speed at Shuffled: F(2,24) = 8.92, p = .001, η_p^2 = .43<br>Presentation speed at Constant: F(2,24) = 8.34, p = .002, η_p^2 = .41<br><br>Prosody Type at 3 LPS: F(2,24) = 8.24, p = .002, η_p^2 = .41<br>Prosody Type at 6 LPS: F(2,24) = .51, p = .607, η_p^2 = .04<br>Prosody Type at 12 LPS: F(2,24) = 1.80, p = .187, η_p^2 = .13	**	Shuffled: 3, 12 < 6 LPS<br>Constant: 3 < 6, 12 LPS<br><br>3 LPS: Shuffled, Constant < Recorded
Emotionality	Speed	$F(2,50) = 8.08, p = .001, \eta_p^2 = .24$	**	3, 12 < 6 LPS
	Prosody Type	$F(2,50) = 1.12, p = .334, \eta_p^2 = .04$		
	Speed × Prosody Type	$F(4,100) = 7.35, p < .0001, \eta_p^2 = .23$<br><br>Simple Main Effects<br>Presentation speed at Recorded: F(2,24) = 9.83, p = .001, η_p^2 = .45<br>Presentation speed at Shuffled: F(2,24) = 5.93, p = .008, η_p^2 = .33<br>Presentation speed at Constant: F(2,24) = 8.53, p = .002, η_p^2 = .42<br><br>Prosody Type at 3 LPS: F(2,24) = 13.19, p < .0001, η_p^2 = .52<br>Prosody Type at 6 LPS: F(2,24) = 1.16, p = .330, η_p^2 = .09<br>Prosody Type at 12 LPS: F(2,24) = 1.84, p = .180, η_p^2 = .13	***	Recorded: 12 < 3, 6 LPS<br>Shuffled: 12 < 6 LPS<br>Constant: 3 < 6 LPS<br><br>3 LPS: Constant < Recorded

Supplementary Table 6. Results of ANOVA for Impression of Reading in Experiment 3 (Hearing loss; Telegram).

Impression	Effect	F value, p value, η_p^2 of Main Effects, Interactions, and Simple Main Effects		Multiple Comparison (Bonferroni)
Readability	Speed	$F(2,48) = 3.28, p = .046, \eta_p^2 = .12$	*	–
	Prosody Type	$F(2,48) = 5.66, p = .006, \eta_p^2 = .19$	**	Shuffled, Constant < Recorded
	Speed × Prosody Type	$F(4,96) = 1.42, p = .235, \eta_p^2 = .06$		
Favorability	Speed	$F(2,48) = 5.34, p = .008, \eta_p^2 = .18$	**	3, 12 < 6 LPS
	Prosody Type	$F(2,48) = 3.54, p = .037, \eta_p^2 = .13$	*	Constant < Recorded
	Speed × Prosody Type	$F(4,96) = .01, p = 1.000, \eta_p^2 = .00$		
Emotionality	Speed	$F(2,48) = 9.09, p < .0001, \eta_p^2 = .28$	**	12 < 3, 6 LPS
	Prosody Type	$F(2,48) = 4.38, p = .018, \eta_p^2 = .15$	*	Constant < Recorded
	Speed × Prosody Type	$F(2.71,65.12) = .58, p = .681, \eta_p^2 = .02$		

Supplementary Table 7. Results of ANOVA for Impression of Reading in Experiment 3 (Hearing loss; Weather Forecast).

Impression	Effect	F value, p value, η_p^2 of Main Effects, Interactions, and Simple Main Effects		Multiple Comparison (Bonferroni)
Readability	Speed	$F(2,32) = 16.19, p < .0001, \eta_p^2 = .50$	***	3 < 12 < 6LPS
	Prosody Type	$F(2,32) = 7.11, p = .003, \eta_p^2 = .31$	**	Shuffled < Recorded, Constant
	Speed × Prosody Type	$F(4,64) = .71, p = .586, \eta_p^2 = .04$		
Favorability	Speed	$F(2,32) = 15.99, p < .0001, \eta_p^2 = .50$	***	3 < 6, 12 LPS
	Prosody Type	$F(2,32) = 5.10, p = .012, \eta_p^2 = .24$	*	Shuffled < Constant
	Speed × Prosody Type	$F(4,64) = 1.49, p = .215, \eta_p^2 = .09$		
Emotionality	Speed	$F(2,32) = 8.27, p = .001, \eta_p^2 = .34$	**	12 < 6LPS
	Prosody Type	$F(2,32) = .81, p = .453, \eta_p^2 = .05$		
	Speed × Prosody Type	$F(4,64) = 1.60, p = .184, \eta_p^2 = .09$		

Supplementary Table 8. Results of ANOVA for Impression of Reading in Experiment 3 (Hearing loss; Earthquake Warning).

Impression	Effect	*F* value, *p* value, η_p^2 of Main Effects, Interactions, and Simple Main Effects	Multiple Comparison (Bonferroni)
Readability	Speed	$F(1.38,22.13) = 22.50, p < .0001, \eta_p^2 = .58$ ***	3 < 6, 12 LPS
	Prosody Type	$F(2,48) = 14.92, p < .0001, \eta_p^2 = .48$ ***	Shuffled < Recorded, Constant
	Speed × Prosody Type	$F(4,96) = 2.58, p = .046, \eta_p^2 = .14$ *<br><br>Simple Main Effects<br>Presentation speed at Recorded: F(2,15) = 9.18, p = .002, $\eta_p^2 = .55$<br>Presentation speed at Shuffled: F(2,15) = 18.91, p < .0001, $\eta_p^2 = .72$<br>Presentation speed at Constant: F(2,15) = 11.69, p = .001, $\eta_p^2 = .61$<br><br>Prosody Type at 3 LPS: F(2,15) = 6.85, p = .008, $\eta_p^2 = .48$<br>Prosody Type at 6 LPS: F(2,15) = 3.72, p = .049, $\eta_p^2 = .33$<br>Prosody Type at 12 LPS: F(2,15) = .85, p = .446, $\eta_p^2 = .10$	Recorded: 3 < 6, 12 LPS<br>Shuffled: 3 < 6 < 12 LPS<br>Constant: 3 < 6, 12 LPS<br><br>3 LPS: Shuffled < Recorded, Constant<br>6 LPS: Shuffled < Recorded
Favorability	Speed	$F(1.37,21.86) = 26.032\ p < .0001, \eta_p^2 = .62$ ***	3 < 6 < 12 LPS
	Prosody Type	$F(2,32) = 8.60, p = .001, \eta_p^2 = .35$ **<br><br>Simple Main Effects<br>Presentation speed at Recorded: F(2,15) = 12.67, p = .001, $\eta_p^2 = .63$<br>Presentation speed at Shuffled: F(2,15) = 12.96, p = .001, $\eta_p^2 = .63$<br>Presentation speed at Constant: F(2,15) = 4.48, p = .030, $\eta_p^2 = .37$<br><br>Prosody Type at 3 LPS: F(2,15) = 8.52, p = .003, $\eta_p^2 = .53$<br>Prosody Type at 6 LPS: F(2,15) = 16.47, p < .0001, $\eta_p^2 = .69$<br>Prosody Type at 12 LPS: F(2,15) = 2.39, p = .126, $\eta_p^2 = .24$	Recorded: 3 < 6, 12 LPS<br><br>Shuffled, Constant: 3 < 6 < 12 LPS<br><br>3, 6 LPS: Shuffled < Recorded, Constant
	Speed × Prosody Type	$F(4,64) = 1.03, p = .398, \eta_p^2 = .06$	
Emotionality	Speed	$F(1.43,22.94) = 1.24, p = .303, \eta_p^2 = .07$	
	Prosody Type	$F(2,48) = 4.23, p = .023, \eta_p^2 = .21$ *	–
	Speed × Prosody Type	$F(4,64) = 1.36, p = .260, \eta_p^2 = .08$	

1.3 Sensorineural Hearing Loss from birth and not from birth.

Supplementary Figs. 9-12 and Supplementary Tables 9-12 show the results of sensorineural hearing loss participants from birth in Experiment 3 for the four text types and three impression types separately. Supplementary Figs. 13-16 show the results of sensorineural hearing loss not by birth for Experiment 3. ANOVAs were not conducted because of the small number of participants that were not hearing loss by birth.

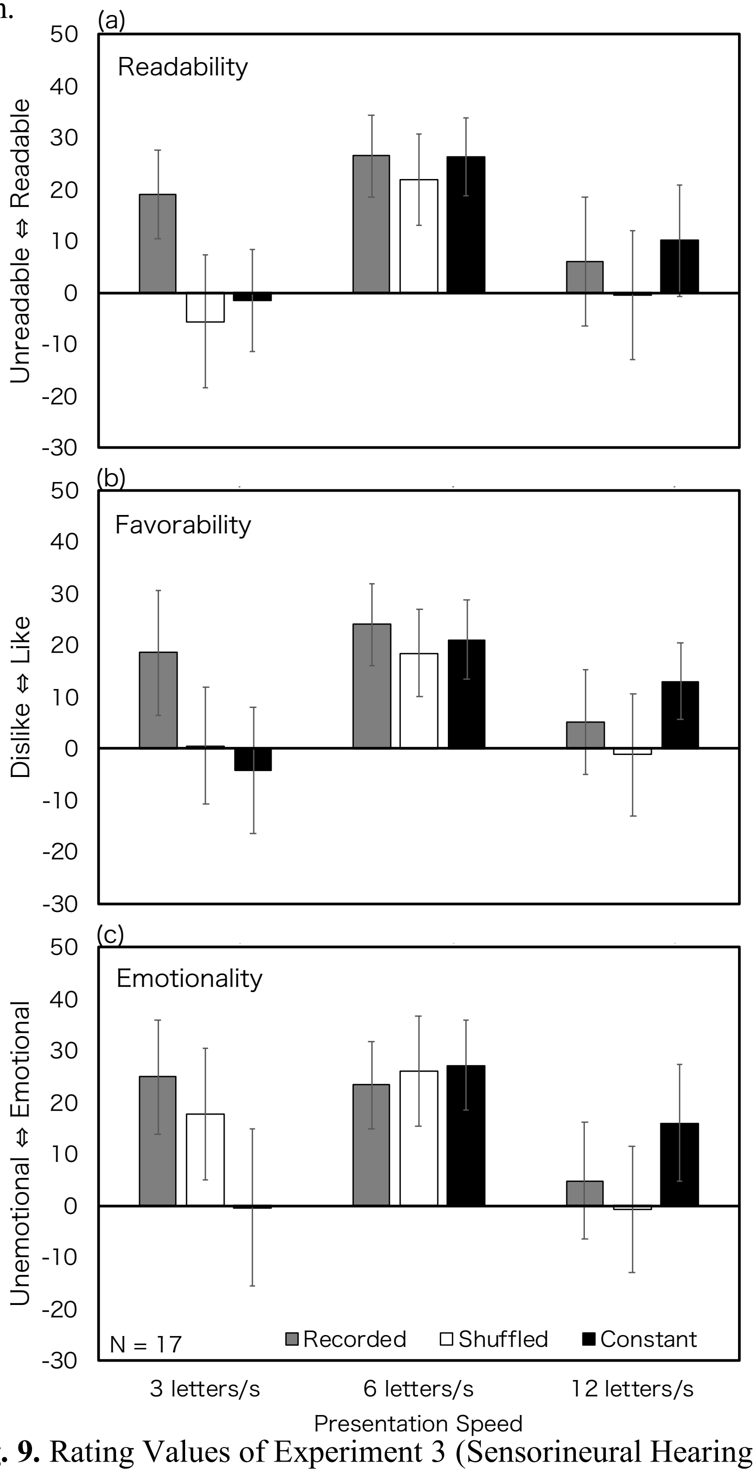

Supplementary Fig. 9. Rating Values of Experiment 3 (Sensorineural Hearing loss from birth; Thank You)

Error bars show 95 % confidential intervals.

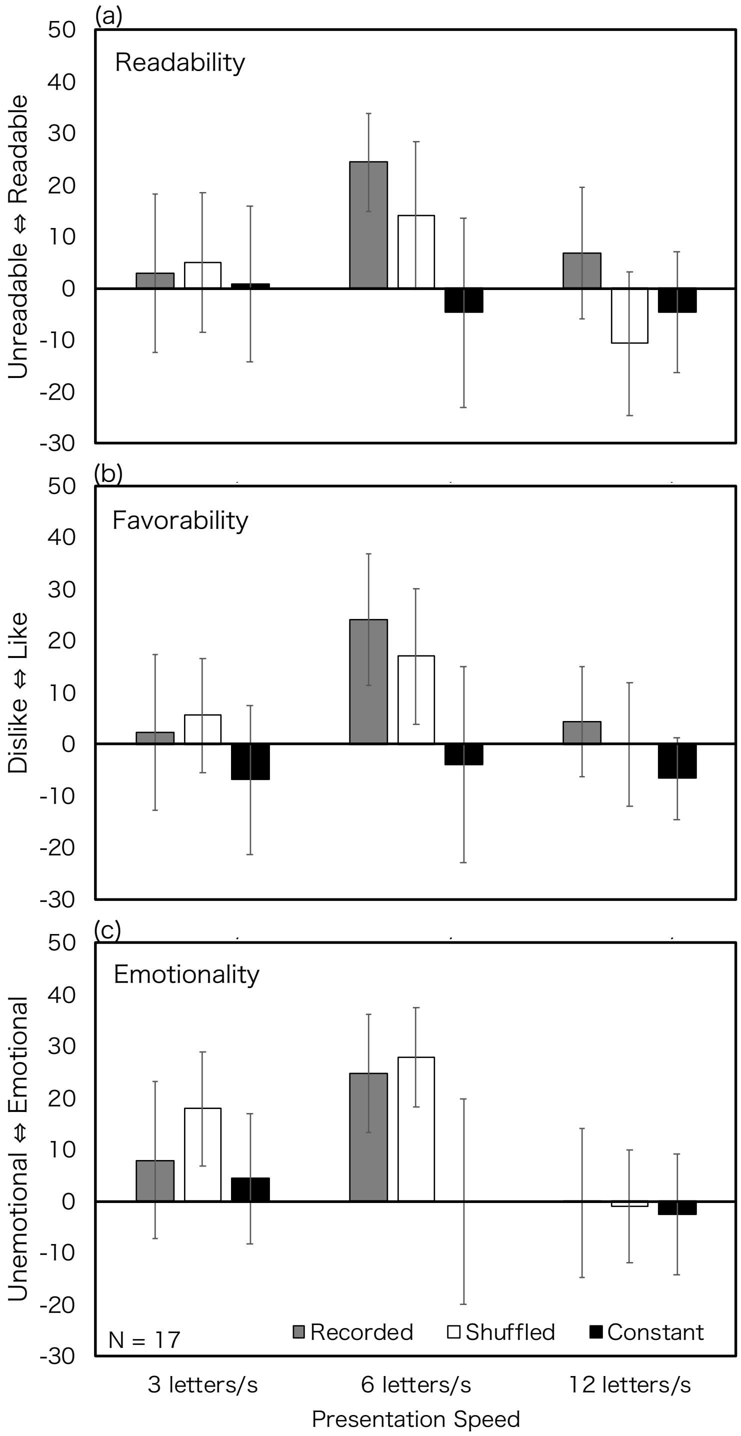

Supplementary Fig. 10. Rating Values of Experiment 3 (Sensorineural Hearing loss from birth; Telegram)

Error bars show 95% confidential intervals.

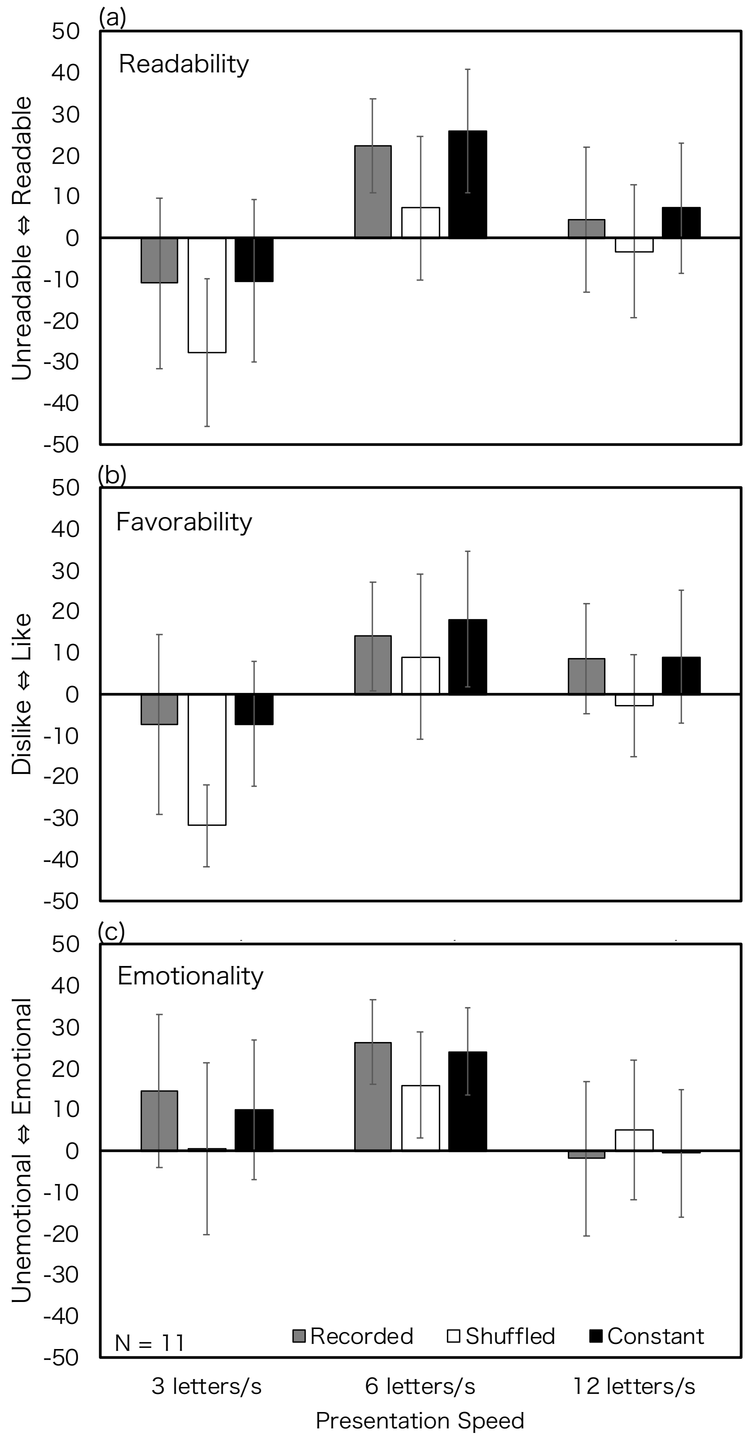

Supplementary Fig. 11. Rating Values of Experiment 3 (Sensorineural Hearing loss from birth; Weather Forecast)

Error bars show 95% confidential intervals.

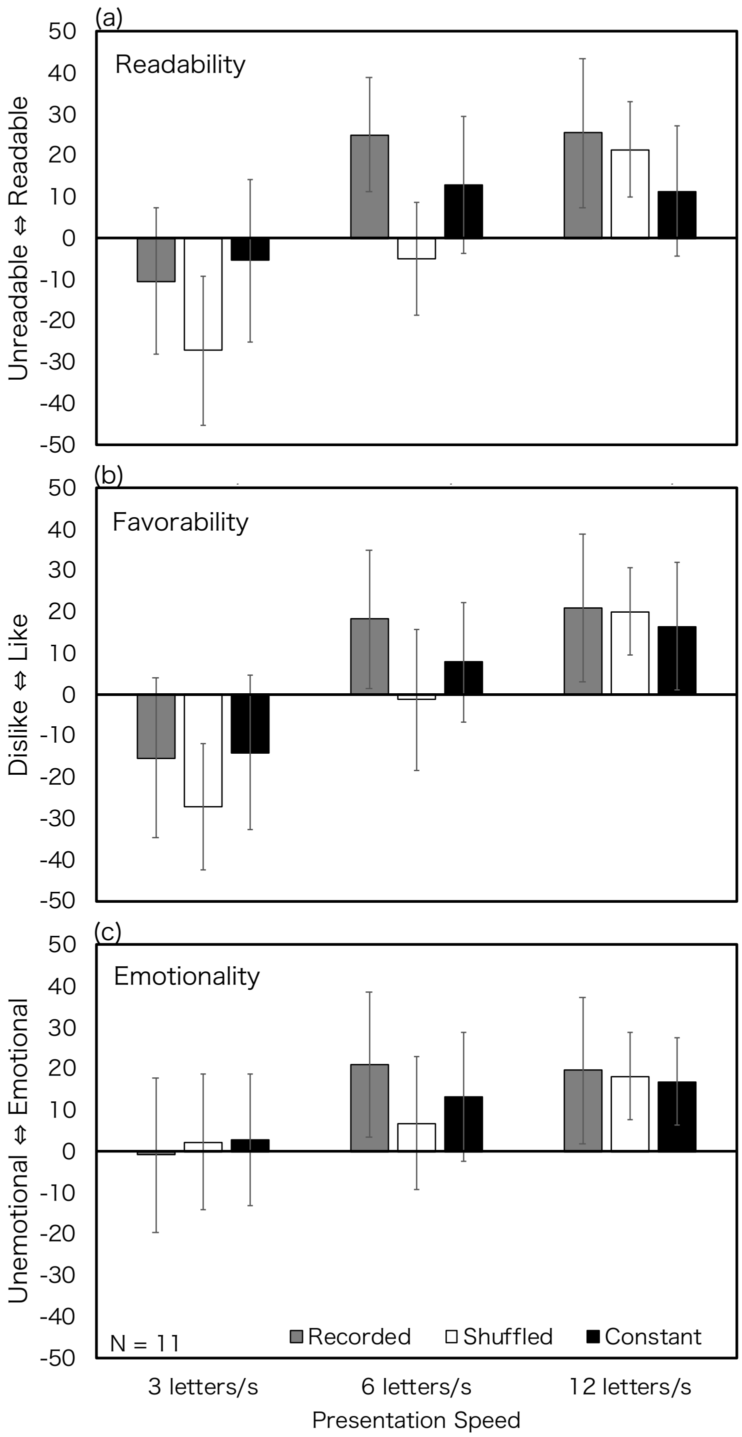

Supplementary Fig. 12. Rating Values of Experiment 3 (Sensorineural Hearing loss from birth; Earthquake Warning)

Error bars show 95% confidential intervals.

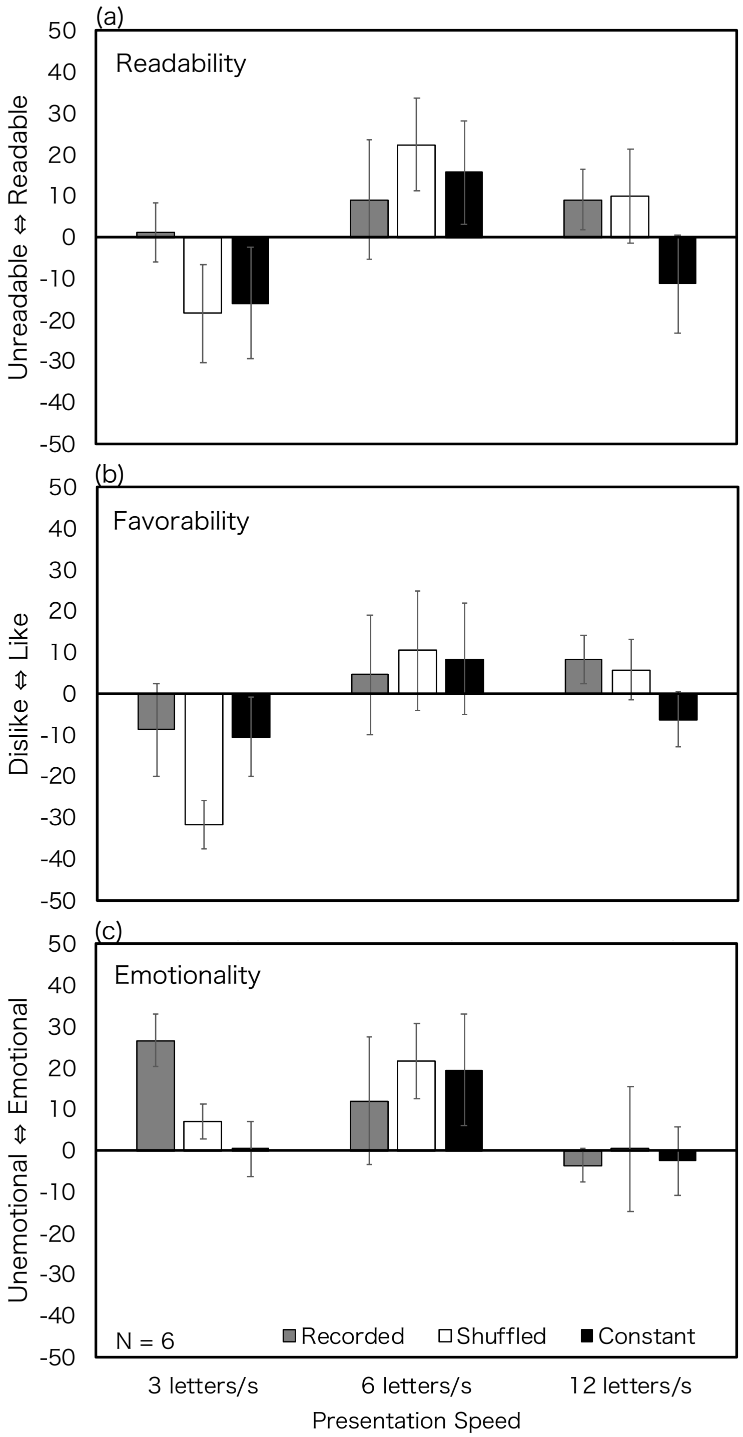

Supplementary Fig. 13. Rating Values of Experiment 3 (Sensorineural Hearing loss not from birth; Thank you)

Error bars show 95% confidential intervals.

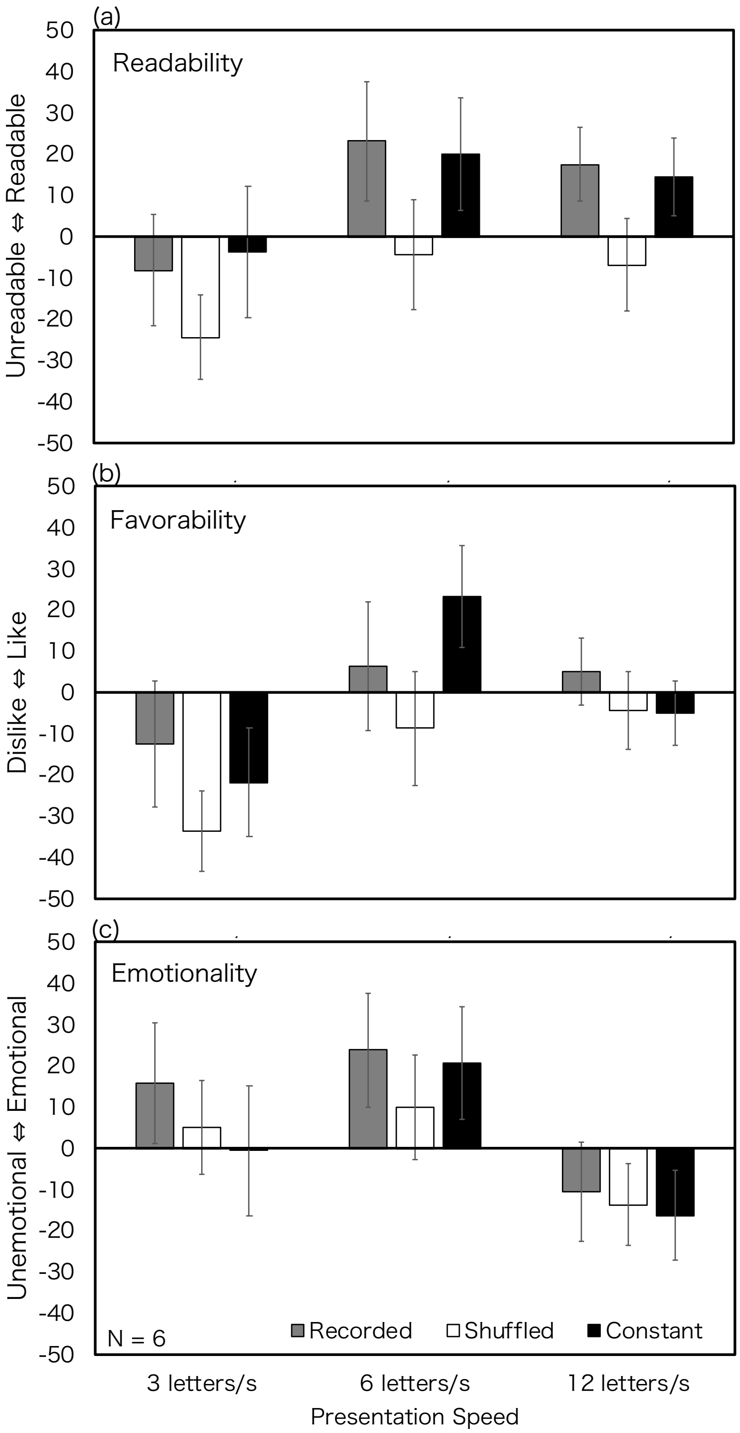

Supplementary Fig. 14. Rating Values of Experiment 3 (Sensorineural Hearing loss not from birth; Telegram)

Error bars show 95% confidential intervals.

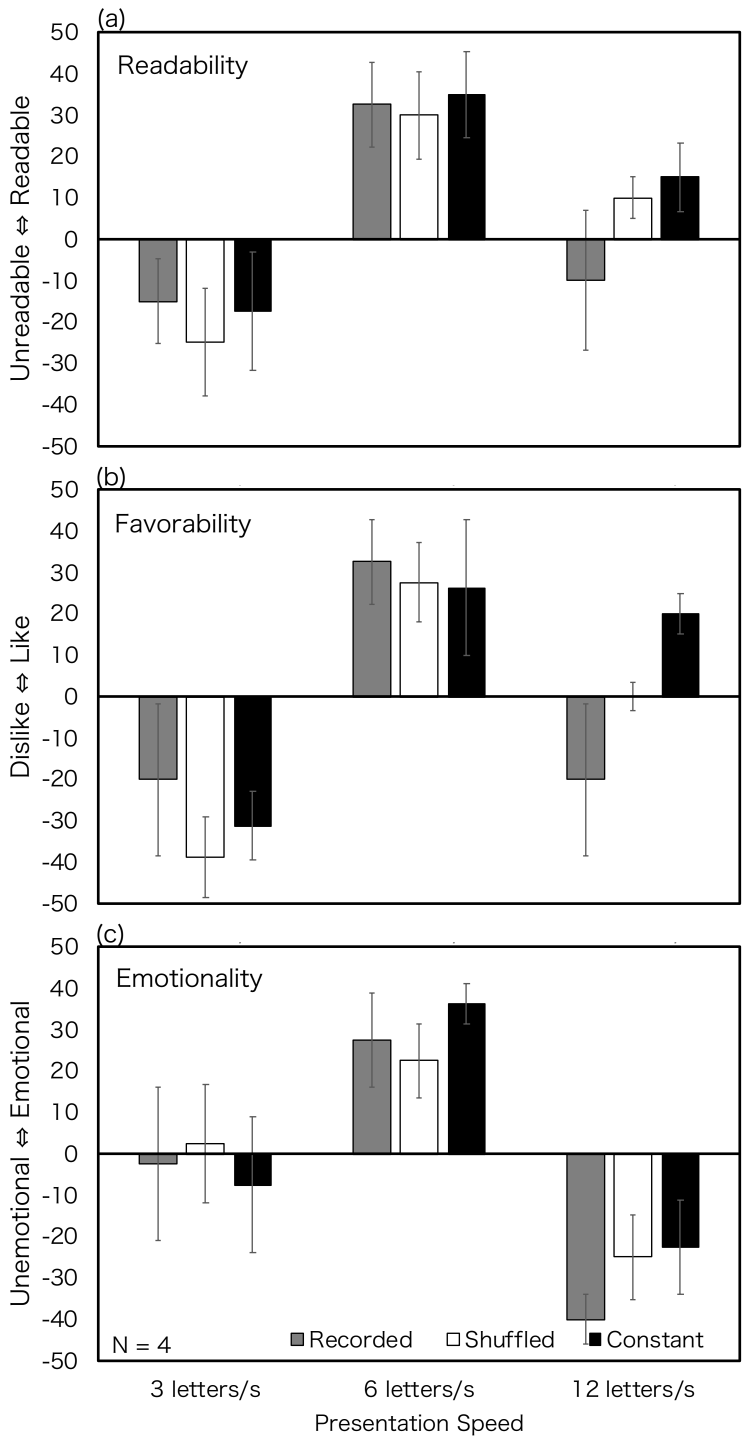

Supplementary Fig. 15. Rating Values of Experiment 3 (Sensorineural Hearing loss not from birth; Weather Forecast)

Error bars show 95% confidential intervals.

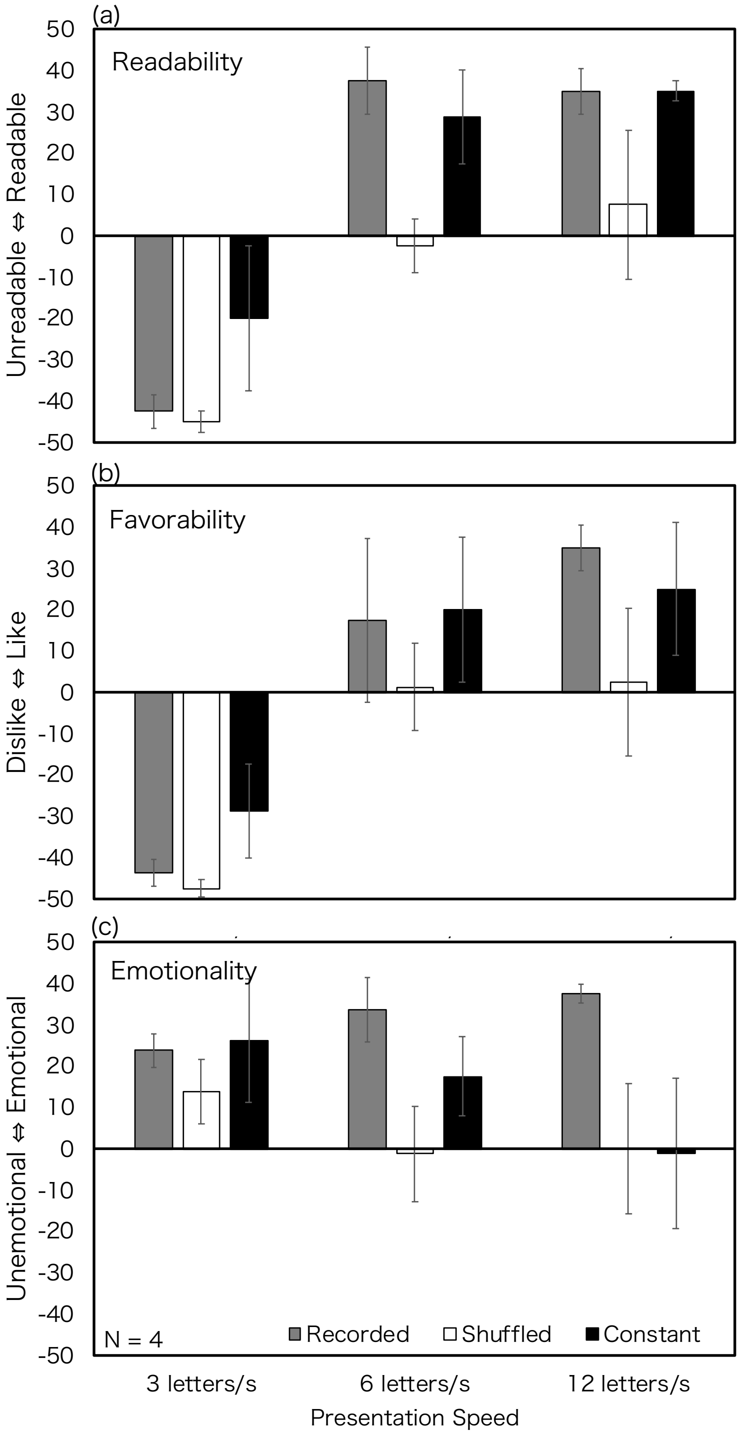

Supplementary Fig. 16. Rating Values of Experiment 3 (Sensorineural Hearing loss not from birth; Earthquake Warning)

Error bars show 95% confidential intervals.

Supplementary Table 9. Results of ANOVA for Impression of Reading in Experiment 3 (Sensorineural Hearing Loss from Birth; Thank you).

Impression	Effect	*F* value, *p* value, η_p^2 of Main Effects, Interactions, and Simple Main Effects		Multiple Comparison (Bonferroni)
Readability	Speed	$F(2,32) = 21.11$, $p < .0001$, $\eta_p^2 = .57$	***	
	Prosody Type	$F(2,32) = 5.41$, $p = .009$, $\eta_p^2 = .25$	**	
	Speed × Prosody Type	$F(4,64) = 4.05$, $p = .005$, $\eta_p^2 = .20$<br><br>Simple Main Effects<br>Presentation speed at Recorded: F(2,15) = 4.55, p = .028, η_p^2 = .38<br>Presentation speed at Shuffled: F(2,15) = 12.13, p = .001, η_p^2 = .62<br>Presentation speed at Constant: F(2,15) = 18.95, p < .0001, η_p^2 = .72<br><br>Prosody Type at 3 LPS: F(2,15) = 7.53, p = .005, η_p^2 = .50<br>Prosody Type at 6 LPS: F(2,15) = .73, p = .496, η_p^2 = .09<br>Prosody Type at 12 LPS: F(2,15) = 1.87, p = .189, η_p^2 = .20	**	Recorded: 12 < 6 LPS<br><br>Shuffled: 3, 12 < 6 LPS<br><br>Constant: 3, 12 < 6 LPS<br><br>3 LPS: Shuffled, Constant < Recorded
Favorability	Speed	$F(2,32) = 10.74$, $p < .0001$, $\eta_p^2 = .40$	***	
	Prosody Type	$F(2,32) = 3.962$ $p = .029$, $\eta_p^2 = .10$	*	
	Speed × Prosody Type	$F(4,64) = 5.04$, $p = .001$, $\eta_p^2 = .24$<br><br>Simple Main Effects<br>Presentation speed at Recorded: F(2,15) = 7.43, p = .006, η_p^2 = .50<br>Presentation speed at Shuffled: F(2,15) = 9.25, p = .002, η_p^2 = .55<br>Presentation speed at Constant: F(2,15) = 7.65, p = .005, η_p^2 = .50<br><br>Prosody Type at 3 LPS: F(2,15) = 7.07, p = .007, η_p^2 = .49<br>Prosody Type at 6 LPS: F(2,15) = 1.00, p = .390, η_p^2 = .12<br>Prosody Type at 12 LPS: F(2,15) = 2.24, p = .141, η_p^2 = .23	**	Recorded: 12 < 6 LPS<br><br>Shuffled: 3, 12 < 6 LPS<br><br>Constant: 3 < 6, 12 LPS<br><br>3 LPS: Shuffled, Constant < Recorded
Emotionality	Speed	$F(2,32) =10.39$, $p < .0001$, $\eta_p^2 = .39$	***	
	Prosody Type	$F(2,32) = .58$, $p = .568$, $\eta_p^2 = .04$		
	Speed × Prosody Type	$F(2.42,38.77) = 6.091$ $p = .003$, $\eta_p^2 = .28$<br><br>Simple Main Effects<br>Presentation speed at Recorded: F(2,15) = 5.63, p = .015, η_p^2 = .43<br>Presentation speed at Shuffled: F(2,15) = 8.33, p = .004, η_p^2 = .53<br>Presentation speed at Constant: F(2,15) = 14.08, p < .0001, η_p^2 = .65<br><br>Prosody Type at 3 LPS: F(2,15) = 7.15, p = .007, η_p^2 = .49<br>Prosody Type at 6 LPS: F(2,15) = .43, p = .661, η_p^2 = .05<br>Prosody Type at 12 LPS: F(2,15) = 2.59, p = .108, η_p^2 = .26	**	Recorded, Shuffled: 12 < 3, 6 LPS<br><br>Constant: 3, 12 < 6 LPS<br><br>3 LPS: Constant < Recorded

Supplementary Table 10. Results of ANOVA for Impression of Reading in Experiment 3 (Sensorineural Hearing Loss from Birth; Telegram).

Impression	Effect	F value, p value, η_p^2 of Main Effects, Interactions, and Simple Main Effects	Multiple Comparison (Bonferroni)
Readability	Speed	$F(1.47,23.44) = 2.897, p = .089, \eta_p^2 = .15$	
	Prosody Type	$F(1.27,20.35) = 4.25, p = .044, \eta_p^2 = .21$ *	Constant < Recorded
	Speed × Prosody Type	$F(4,64) = 1.99, p = .106, \eta_p^2 = .11$	
Favorability	Speed	$F(1.25,20.05) = 3.81, p = .057, \eta_p^2 = .19$	
	Prosody Type	$F(2,32) = 9.02, p = .001, \eta_p^2 = .36$ **	Constant < Recorded, Shuffled
	Speed × Prosody Type	$F(4,64) = .88, p = .482, \eta_p^2 = .05$	
Emotionality	Speed	$F(2,32) = 8.06, p = .001, \eta_p^2 = .34$ **	12 < 3, 6 LPS
	Prosody Type	$F(2,32) = 4.62, p = .017, \eta_p^2 = .22$ *	Constant < Recorded
	Speed × Prosody Type	$F(4,64) = 1.93, p = .\ 116, \eta_p^2 = .11$	

Supplementary Table 11. Results of ANOVA for Impression of Reading in Experiment 3 (Sensorineural Hearing Loss from Birth; Forecast).

Impression	Effect	F value, p value, η_p^2 of Main Effects, Interactions, and Simple Main Effects	Multiple Comparison (Bonferroni)
Readability	Speed	$F(2,20) = 7.53, p = .004, \eta_p^2 = .43$ **	3 < 6 LPS
	Prosody Type	$F(2,20) = 5.96, p = .009, \eta_p^2 = .37$ **	Shuffled < Recorded, Constant
	Speed × Prosody Type	$F(4,40) = .22, p = .923, \eta_p^2 = .02$	
Favorability	Speed	$F(2,20) = .17, p = .847, \eta_p^2 = .02$	
	Prosody Type	$F(2,20) = .12, p = .887, \eta_p^2 = .01$	
	Speed × Prosody Type	$F(4,40) = 3.95, p = .009, \eta_p^2 = .28$ **<br><br>Simple Main Effects<br>Presentation speed at Recorded: F(2,9) = 2.44, p = .142, $\eta_p^2 = .35$<br>Presentation speed at Shuffled: F(2,9) = 1.95, p = .198, $\eta_p^2 = .30$<br>Presentation speed at Constant: F(2,9) = 1.07, p = .383, $\eta_p^2 = .19$<br><br>Prosody Type at 3 LPS: F(2,9) = 5.12, p = .033, $\eta_p^2 = .53$<br>Prosody Type at 6 LPS: F(2,9) = 3.05, p = .098, $\eta_p^2 = .40$<br>Prosody Type at 12 LPS: F(2,9) = 2.47, p = .140, $\eta_p^2 = .35$	3 LPS: Recorded < Shuffled
Emotionality	Speed	$F(1.22,12.15) = 3.46, p = .082, \eta_p^2 = .26$	
	Prosody Type	$F(2,20) = 1.16, p = .334, \eta_p^2 = .10$	
	Speed × Prosody Type	$F(2.19,21.85) = 1.61, p = .223, \eta_p^2 = .14$	

Supplementary Table 12. Results of ANOVA for Impression of Reading in Experiment 3 (Sensorineural Hearing Loss from Birth; Earthquake Warning).

Impression	Effect	*F* value, *p* value, η_p^2 of Main Effects, Interactions, and Simple Main Effects	Multiple Comparison (Bonferroni)
Readability	Speed	$F(2,20) = 9.78$, $p = .001$, $\eta_p^2 = .49$ **	
	Prosody Type	$F(2,20) = 5.85$, $p = .010$, $\eta_p^2 = .37$ *	
	Speed × Prosody Type	$F(4,40) = 4.18$, $p = .006$, $\eta_p^2 = .30$ **<br><br>Simple Main Effects<br>Presentation speed at Recorded: F(2,9) = 6.86, p = .015, $\eta_p^2 = .60$<br>Presentation speed at Shuffled: F(2,9) = 8.48, p = .009, $\eta_p^2 = .65$<br>Presentation speed at Constant: F(2,9) = 1.56, p = .263, $\eta_p^2 = .26$<br><br>Prosody Type at 3 LPS: F(2,9) = 5.37, p = .029, $\eta_p^2 = .54$<br>Prosody Type at 6 LPS: F(2,9) = 9.19, p = .007, $\eta_p^2 = .67$<br>Prosody Type at 12 LPS: F(2,9) = 1.64, p = .247, $\eta_p^2 = .27$	Recorded: 3 < 6, 12 LPS<br><br>Shuffled, Constant: 3, 6 < 12 LPS<br><br>3, 6 LPS: Shuffled < Recorded
Favorability	Speed	$F(2,20) = 18.35$, $p < .0001$, $\eta_p^2 = .65$ ***	3 < 6, 12 LPS
	Prosody Type	$F(2,20) = 3.52$, $p = .049$, $\eta_p^2 = .26$ *	–
	Speed × Prosody Type	$F(4,40) = 1.88$, $p = .133$, $\eta_p^2 = .16$	
Emotionality	Speed	$F(2,20) = 3.23$, $p = .061$, $\eta_p^2 = .24$	
	Prosody Type	$F(2,20) = .94$, $p = .406$, $\eta_p^2 = .09$	
	Speed × Prosody Type	$F(2.251,22.507) = .80$, $p = .475$, $\eta_p^2 = .07$	
